# Assessing the transition of COVID-19 burden towards the young population while vaccines are rolled out in China*

**DOI:** 10.1080/22221751.2022.2063073

**Published:** 2022-04-25

**Authors:** Jun Cai, Juan Yang, Xiaowei Deng, Cheng Peng, Xinhua Chen, Qianhui Wu, Hengcong Liu, Juanjuan Zhang, Wen Zheng, Junyi Zou, Zeyao Zhao, Marco Ajelli, Hongjie Yu

**Affiliations:** a School of Public Health, Fudan University, Key Laboratory of Public Health Safety, Ministry of Education, Shanghai, People's Republic of China; b Department of Infectious Diseases, Huashan Hospital, School of Public Health, Fudan University, Shanghai, People's Republic of China; c Shanghai Institute of Infectious Disease and Biosecurity, Fudan University, Shanghai, People’s Republic of China; d Laboratory for Computational Epidemiology and Public Health, Department of Epidemiology and Biostatistics, Indiana University School of Public Health, Bloomington, IN, USA

**Keywords:** Novel coronavirus disease 2019, Delta variant, vaccination, transition of disease burden, children, China

## Abstract

SARS-CoV-2 infection causes most cases of severe illness and fatality in older age groups. Over 92% of the Chinese population aged ≥12 years has been fully vaccinated against COVID-19 (albeit with vaccines developed against historical lineages). At the end of October 2021, the vaccination programme has been extended to children aged 3–11 years. Here, we aim to assess whether, in this vaccination landscape, the importation of Delta variant infections could shift COVID-19 burden from adults to children. We developed an age-structured susceptible-infectious-removed model of SARS-CoV-2 transmission to simulate epidemics triggered by the importation of Delta variant infections and project the age-specific incidence of SARS-CoV-2 infections, cases, hospitalizations, intensive care unit admissions, and deaths. In the context of the vaccination programme targeting individuals aged ≥12 years, and in the absence of non-pharmaceutical interventions, the importation of Delta variant infections could have led to widespread transmission and substantial disease burden in mainland China, even with vaccination coverage as high as 89% across the eligible age groups. Extending the vaccination roll-out to include children aged 3–11 years (as it was the case since the end of October 2021) is estimated to dramatically decrease the burden of symptomatic infections and hospitalizations within this age group (39% and 68%, respectively, when considering a vaccination coverage of 87%), but would have a low impact on protecting infants. Our findings highlight the importance of including children among the target population and the need to strengthen vaccination efforts by increasing vaccine effectiveness.

## Introduction

The coronavirus disease 2019 (COVID-19) pandemic is still raging worldwide. The highly contagious Delta variant (B.1.617.2) of the severe acute respiratory syndrome coronavirus 2 (SARS-CoV-2) was the dominant strain across the world from June 2021 to November–December 2021 [1]. Afterwards, the Omicron variant has become dominant in most countries and the global circulation of both Delta and Omicron variants has led to multiple waves of COVID-19 cases worldwide, including in areas with high vaccination coverage [2].

A clear positive correlation has been shown between severe illness and fatality from SARS-CoV-2 infection and age [3,4]. COVID-19 vaccines are highly efficacious in preventing severe illness [5,6]. Due to individual choices and age-prioritized vaccination strategies adopted by most countries [7], vaccine coverage tends to be higher in older adults [8,9]. However, since the summer of 2021, while the Delta variant was spreading, the number of COVID-19 cases and hospitalizations had trended upwards in many countries with an increasing fraction of children and adolescents [10–12].

China has been able to avoid the widespread local transmission of SARS-CoV-2 since April 2020. However, the importations of Delta variant infections caused dozens of local outbreaks [13], the largest of which occurred in Xi’an city and led to 2050 reported cases and a city-wide lockdown [14]. Similar to what was observed in other countries, the Delta surge was characterized by a high proportion of infections in children (eg 77/226 reported cases in Putian city, China were younger than 12 years) [15].

In China, two inactivated COVID-19 vaccines have obtained emergency use approval for administration in individuals aged ≥3 years; as of March 2022, no vaccine has received approval for use in children aged ≤2 years. To prevent the spread of infection in younger age groups, China extended the vaccination programme to include adolescents aged 12–17 years in July 2021, then to include children aged 3–11 years around the end of October 2021 (Figure S2). As of 29 October 2021, 2.26 billion and 3.53 million doses were administered to 12+ years and 3–11 years group, respectively, corresponding to 92% and 1% of these two target populations [16].

The vaccines used in mainland China were developed using historical SARS-CoV-2 lineages and have proven to be highly effective in protecting against severe illness caused by the Delta variant [5,17–20]. However, their effectiveness in preventing Delta infections appears to be reduced [17]. Given the lower vaccination coverage in children as compared to adults, it is legit to ask whether an epidemic caused by the importation of Delta variant infections could shift COVID-19 burden towards younger age groups and how this would affect the return to normal. To fill this gap, we developed an age-structured SARS-CoV-2 transmission model to project age-specific epidemiological outcomes, should an epidemic start to unfold. In particular, the model is used to simulate alternative vaccination strategies and allows tracking the number of infections, cases, hospitalizations, intensive care unit (ICU) admissions, and deaths by age.

## Methods

### SARS-CoV-2 transmission and vaccination model

We developed an age-structured (16 age groups, Table S2) stochastic compartmental susceptible-infectious-removed (SIR) model to simulate the transmission of SARS-CoV-2 (Delta variant) and assess the health impact of age-targeted vaccination campaigns (Figure S1). Detailed information about the model and adopted parameters are described in Section 1.1 of Supplementary Materials and Methods. A summary is presented here.

The model is calibrated to represent the Chinese population and considers the age-mixing patterns quantified prior to the COVID-19 pandemic [21]. Based on contact tracing data in the Hunan province of China [22], children aged under 15 years were found to have a lower susceptibility to SARS-CoV-2 infection than other age groups, which was confirmed by several independent studies [23] (Table S1). A sensitivity analysis considering homogeneous susceptibility across age groups is presented in Figure S3. As of November 2020, the entire population of China virtually remained susceptible to SARS-CoV-2 after containment of the first epidemic wave of COVID-19 [24], and thus the model was initialized with a fully susceptible population. Simulations were seeded with 40 imported infections [25] on 1 December 2021 and run forward for six months. As sensitivity analyses, we considered also 10 and 20 seeds (Table S1). We considered a basic reproductive number *R_0_* = 6 for the Delta variant [26]. A study conducted in the UK and one in China estimated a shorter generation time than for historical lineages [27,28]. In the baseline analysis, we set the average generation time of the Delta variant to be 4.6 days [28]. However, another analysis conducted in Singapore found no significant difference [29]. We thus considered a longer generation time of seven days, in line with estimates for the original lineages [30], in a sensitivity analysis (Table S1).

### Vaccination strategy and vaccine effectiveness

We considered two vaccination strategies: (1) vaccination of individuals aged ≥12 years (here referred to as the “adults + adolescents” vaccination strategy), in agreement with the vaccination programme in place in China until mid-October 2021; (2) as in strategy (1), but the target population was extended to include children aged 3–11 years, as it was the case in China starting from 28 October 2021 – this strategy is referred to here as the “adults + adolescents + children” vaccination strategy. We considered that susceptible individuals only are eligible for vaccination, mimicking the programme implemented in China. Booster doses were not considered in this study. Moreover, we accounted for individuals with contraindications against vaccination and pregnant women as they were excluded from the Chinese vaccination programme (Table S2) [26]. We simulated the daily distribution of vaccine doses according to the observed vaccination capacity (see Section 1.2 in Supplementary Materials and Methods for details).

We considered a two-dose vaccine with a 21-day interval between doses. Fourteen days after the second dose, the vaccine efficacy in protecting against an infection caused by the Delta variant was set at 51.8% [17]. We considered different vaccine effectiveness (VE) against each of the following clinical endpoints: infection, symptomatic disease, hospitalization, ICU admission, and death (see Table 1). We further explored a range of higher VE values for sensitivity analyses (Figure S9). We considered VE to be homogeneous across age groups [31–33]. We considered a “leaky” vaccine in which all vaccinated individuals are exposed to a lower risk of infection, which is 1-VE times that of non-vaccinated individuals [34]. We assumed that both the vaccine and natural infection confer protection for longer than the study period (ie one year) [35].

**Table 1.** Parameters regulating the transmissibility, vaccine effectiveness, and COVID-19 burden.

Parameter description	Estimated value for the Delta variant
*Epidemiology*	
Basic reproduction number (*R_0_*)	6 [26]
*Vaccine effectiveness*	
Against infection (%) (ϵ)	51.8 (20.3, 83.2) [17]
Against symptomatic disease (%) ($\epsilon^{symp}$)	60.4 (31.8, 88.9) [17,18,20]
Against hospitalization (%) ($\epsilon^{hosp}$)	82.6 (82.0, 83.1) [5]^a^
Against ICU admission (%) ($\epsilon^{icu}$)	83.6 (82.8, 84.3)^b^
Against death (%) ($\epsilon^{death}$)	83.6 (82.8, 84.3) [5,20]^a^
*Disease burden*^c^	
Age-specific proportion of unvaccinated infections that develop symptoms for the wild-type ($r_{a,WT}^{symp}$)	39.79%, 50.51%, 67.52%, 66.17%, and 64.6% for 0–19, 20–39, 40–59, and ≥60 years [36]
Age-specific proportion of unvaccinated infections that require hospitalization for the wild-type ($r_{a,WT}^{hosp}$)	0.40%, 0.28%, 0.76%, 1.31%, 2.30%, 3.31%, 4.70%, 8.27% and 10.72% separately for 0–9, 10–19, 20–29, 30–39, 40–49, 50–59, 60–69, 70–79 and ≥80 years [36,37]
Age-specific proportion of unvaccinated infections that require ICU for the wild-type ($r_{a,WT}^{icu}$)	0.0091%, 0.0077%, 0.0123%, 0.0446%, 0.1353%, 0.3651%, 0.7685%, 1.3233%, 1.7930%, and 0.6009% separately for 0–4, 5–9, 10–19, 20–29, 30–39, 40–49, 50–59, 60–69, 70–79, and ≥80 years [36–38]
Age-specific infection fatality risk of the wild-type among unvaccinated individuals ($r_{a,WT}^{death}$)	0.0025%, 0.0018%, 0.0093%, 0.0162%, 0.0953%, 0.1373%, 0.9265%, 1.6311%, and 2.1139% separately for 0–9, 10–19, 20–29, 30–39, 40–49, 50–59, 60–69, 70–79, and ≥80 years [3,36,37]
Risk ratio of symptomatic infection associated with the Delta variant compared to the wild-type ($\Delta_{WT\to Delta}^{symp}$)	1 [39]^d^
Risk ratio of hospitalization associated with the Delta variant compared to the wild-type ($\Delta_{WT\to Delta}^{hosp}$)	2.78 (1.92, 4.13) [37,40–42]
Risk ratio of ICU admission associated with the Delta variant compared to the wild-type ($\Delta_{WT\to Delta}^{icu}$)	3.17 (1.95, 5.59) [41,43]
Risk ratio of death associated with the Delta variant compared to the wild-type ($\Delta_{WT\to Delta}^{death}$)	2.33 (1.54, 3.31) [41]

^a^ Although the dominant variants of concern detected in Brazil during the study period from 24 February 2020 to 11 November 2021 were the Gamma and Delta variants, we assume that the effectiveness reported in reference [5] applies to the Delta variant.

^b^ We assume that the effectiveness of inactivated COVID-19 vaccines against ICU admission caused by the Delta variant is the same as that against death.

^c^ The risks of different clinical outcomes associated with the Delta variant are expressed as increased risks compared to the wild-type.

^d^ Based on the conclusion that no significant change in reported symptoms associated with the Alpha variant compared to wild-type is found in reference [39], we assume that the probability of developing symptoms by age for the Delta variant is the same as the wild-type.

### Model of COVID-19 burden

The number of infections was produced by the transmission model previously described. To estimate COVID-19 burden, we rescaled the number of infections to obtain estimates of the cumulative incidence of symptomatic cases, hospitalizations, ICU admissions, and deaths under different vaccination strategies using the corresponding age-specific risks of Delta estimated for unvaccinated and vaccinated individuals. The age-specific risks for vaccinated individuals are estimated by adjusting the risks for unvaccinated individuals by conditional VEs against different clinical endpoints given Delta infection. The age-specific risks of disease progression for unvaccinated individuals are based on historical lineages and then corrected for the increased severity of the Delta variant (Table 1 – details reported in Section 1.3 in Supplementary Materials and Methods). Briefly, by integrating studies conducted in China [43], Scotland [40], Canada [41], and England [37,42], we estimated that the increased risk associated with the Delta variant was 1.78 (0.92–3.13) for hospitalization, 2.17 (0.95–4.59) for ICU admission, and 1.33 (0.54–2.31) for death as compared to the original lineage, respectively (Table 1). We also assumed that the age-specific probability of developing symptoms upon Delta infection is similar to that of the historical lineage. This choice was inspired by the finding that no significant difference was found for the Alpha variant as compared to historical lineages [39]. To compare the severity of disease across different age groups, we calculated the rate ratios as the incidence rate per age group dividing the total incidence rate for each health outcome under different vaccination strategies and no vaccination scenario.

### Data analysis

For each scenario, 200 stochastic simulations were performed. The outcomes of these simulations determined the distribution of the cumulative number of symptomatic infections, hospitalizations, ICU admissions, and deaths by age. We defined 95% credible intervals as quantiles 0.025 and 0.975 of the estimated distributions.

## Results

### Baseline scenario

**Figure 1.**
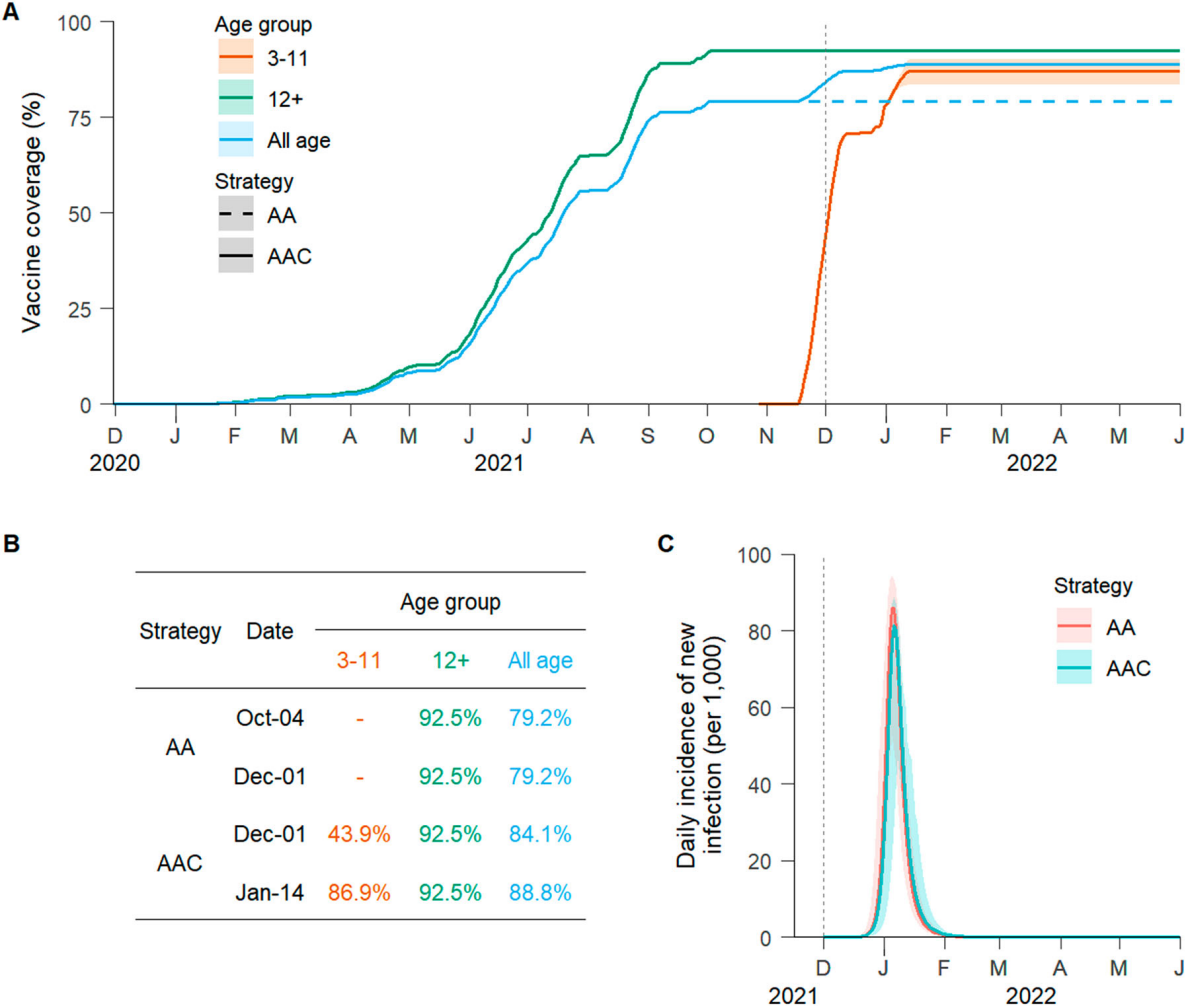
Time series of vaccine coverage and daily incidence of new Delta variant infections. (A) Age-specific vaccine coverage over time under different vaccination strategies (AA = “adults + adolescents” vaccination strategy, AAC = “adults + adolescents + children” vaccination strategy). The vaccination programme was initiated on 30 November 2020 (as first officially reported in China). The vertical dotted lines represent the (simulated) seeding of the infection (1 December 2021). The line corresponds to the mean value, while the shaded area represents the 95% quantile intervals (CI). (B) The table shows the age-specific coverage over time for each vaccination strategy. The vaccine coverage reached the maximum on 4 October 2021 under the AA vaccination strategy and on 14 January 2022 under the AAC vaccination strategy. (C) Simulated daily incidence of new Delta variant infections per 1000 individuals for the two strategies (mean and 95% CI).

The baseline scenario assumes that 40 individuals infected with the Delta variant were introduced in the population on 1 December 2021, and the basic reproduction number *R_0_* was 6 in the absence of interventions and immunity. Given vaccination rates data, the “adults + adolescents” vaccination strategy reached a 92.5% coverage of the target population (79.2% of the total population) by 4 October 2021. While under the “adults + adolescents + children” vaccination strategy, about 43.9% of children aged 3–11 years were fully vaccinated at the assumed time of importation of the Delta variant (ie 1 December 2021), and the coverage of the total population increased to 88.8% (86.9% and 92.5% among 3–11 and 12+ age groups, respectively) by 14 January 2020 (Figure 1(A)).

**Figure 2.**
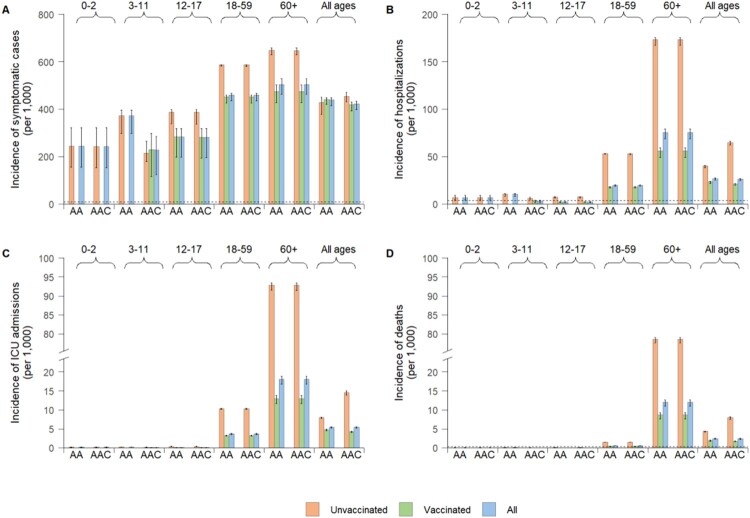
Age profile of estimated disease burden caused by an epidemic of imported Delta variant infections in China. (A) Cumulative number of symptomatic cases per 1000 individuals after six simulated months by vaccination strategy (AA = “adults + adolescents” vaccination strategy, AAC = “adults + adolescents + children” vaccination strategy), vaccination status, and age group. The vaccinated group are those individuals who are administrated with two doses. (B) As (A), but for the incidence of hospitalizations. (C) As (A), but for the incidence of ICU admissions. (D) As (A), but for mortality. The horizontal dotted lines in (A), (B), and (D) represent the rates of symptomatic cases, hospitalizations, and deaths, respectively, of the first pandemic wave of COVID-19 in Wuhan, China [3].

Model simulations suggest that the importation of Delta variant infections in December 2021 could have had the potential to generate a major epidemic wave in the absence of non-pharmaceutical interventions (NPIs). Such an epidemic was estimated to cause 439 (95% CI: 415–447) symptomatic infections, 27 (95% CI: 25–28) hospitalizations, 5.4 (95% CI: 5.2–5.6) ICU admissions, and 2.4 (95% CI: 2.2–2.5) deaths per 1000 individuals over a six-month period (Figures 1(C) and Figure 2). These figures correspond to 7–55 fold the disease burden of the initial epidemic in Wuhan, China [3]. Under the “adults + adolescents” vaccination strategy, 11.2% of symptomatic infections and 5.1% of hospitalizations were estimated to occur in children younger than 12 years (Figure S8), who were ineligible for COVID-19 vaccination until mid-October 2021.

On September 2021, two inactivated COVID-19 vaccines (Sinovac/CoronaVac and Sinopharm/BBIBP-CorV) had been licenced for use in children aged 3–17 years. However, vaccination had not been implemented among children aged 3–11 years until the end of October 2021. Here, we simulated a scenario where the vaccine was offered to children aged 3–11 years (representing 11.0% of the population) from 28 October 2021 (ie the “adults + adolescents + children” vaccination strategy). As compared to the “adults + adolescents” vaccination strategy, the “adults + adolescents + children” vaccination strategy is estimated to marginally reduce COVID-19 burden (4%, 95% CI: 1–9% for symptomatic cases and 3%, 95% CI: 1–8% for hospitalized patients) (Figure 2). Moreover, extending vaccination to children is estimated not to be enough to suppress viral circulation.

**Figure 3.**
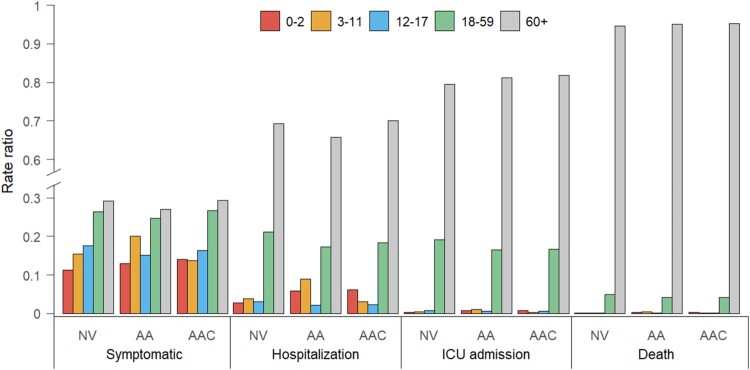
Rate ratios of symptomatic cases, hospitalizations, ICU admissions, and deaths due to Delta variant infections in China by age group and vaccination strategy. NV = no vaccination, AA = “adults + adolescents” vaccination strategy, AAC = “adults + adolescents + children” vaccination strategy.

We used the model to simulate a counterfactual scenario to estimate what would have happened if a vaccination programme had not been implemented. Compared with a no vaccination scenario, the “adults + adolescents” vaccination strategy led to a 15% and 30% increase in the rate ratios of symptomatic infections in children aged 0–2 and 3–11 years, respectively. We recall that the rate ratio is defined as the incidence rate per age group divided by the total incidence rate [44]. A higher increase is observed in the rate ratios of hospitalization for these two population groups: 110% and 137% for children aged 0–2 and 3–11 years, respectively. At the same time, the rate ratios in adults aged ≥60 years are estimated to decrease by 5%. Similar patterns are observed in the rate ratios of ICU admissions and deaths (Figure 3). Extending the vaccination to children aged 3–11 years would dramatically decrease the burden of symptomatic infections and hospitalizations within the same age group by 39% and 68%, respectively (Figure S8). However, due to the strong age-assortativity of contact patterns of this age group (ie individuals aged 3–11 years primarily mix with other individuals of the same age) (Figure S4(A)), extending the vaccination to children does not strongly impact the COVID-19 burden in other age groups (Figure S8). No evident effect is observed on the 0–2-year age group (Figures 3 and S8).

Under either the “adults + adolescents” or “adults + adolescents + children” vaccination strategies, the mean incidence of symptomatic cases among unvaccinated individuals is estimated to be 0.9–1.4 fold that of vaccinated individuals, with larger differences observed in terms of the incidence of severe clinical outcomes (2.0–3.1 fold for hospitalization, 2.1–3.3 fold for ICU admission, and 2.1–3.3 fold for death) (Figure 2).

### Improving VE in the absence/presence of NPIs

In our model, VE in preventing ICU admissions and deaths caused by a Delta variant infection was set at 83.6%, based on real-world VE studies in China and Brazil (Table 1) [5,20]. Should the VE against the infection of the Delta variant be increased to >88% from 51.8% used in the baseline [17], the “adults + adolescents + children” vaccination strategy is estimated to reduce the number of deaths by 97.9% even in the absence of any NPIs, leading to <74,900 deaths over six months, similar to the annual excess respiratory disease deaths associated with influenza in China [45] (Figure S9).

We leveraged the developed model to explore the impact of implementing different levels of NPIs on the COVID-19 burden while the “adults + adolescents + children” vaccination strategy is in place and considering different levels of VE against infection. When VE against infection is set at 51.8% (baseline scenario), stringent NPIs capable of maintaining the net reproduction number *R_e _*< 2.4 are needed, in combination with the vaccination programme, to reduce the yearly COVID-19 death toll to a level similar to that of seasonal influenza [45] (<64,300 deaths). Should a new vaccine with higher (>75%) VE against the infection be adopted, NPIs could be relaxed. Moderate NPIs able to keep *R_e _*< 4.0 in combination with the vaccination programme is estimated to be sufficient to decrease the annual number of deaths to less than 89,000 (Figure S9).

### Sensitivity analyses

In the baseline analysis, age-varying susceptibility to SARS-CoV-2 infection was considered. That is, using adults aged 15–64 years as a reference group, children have a 42% lower risk of infection than adults (Table S1) [22]. No estimates are available for susceptibility to Delta variant infections by age. As such, we performed an analysis assuming the same susceptibility to infection across all age groups. The obtained results are consistent with those obtained in the baseline analysis, with average variations lower than 12% (Figure S3).

Another feature able to shape the epidemiology of COVID-19 is the contact pattern of the population. In the baseline analysis, we used pre-pandemic mixing patterns to represent a situation close to the objective of returning to a pre-COVID-19 pandemic lifestyle [21] (Figure S4A). However, whether mixing patterns will ever return to be the same after the pandemic remains to be seen. As such, we tested the robustness of our findings by considering an alternative contact matrix estimated in Shanghai, China, right after the end of the lockdown in March 2020 [46] (Figure S4B). The obtained results are consistent with those obtained with the pre-pandemic contact matrix, with mean variations in the estimated burden lower than 1% (Figure S5).

We further tested the robustness of our findings by assuming a longer generation time of seven days and less (10 and 20) imported seed infections. The obtained disease burden is consistent with those obtained in the baseline analysis across age groups and vaccination strategies (Figures S6 and S7).

## Discussion

Our modelling study indicates that, under a vaccination programme targeting individuals aged ≥12 years, symptomatic SARS-CoV-2 infections and hospitalization would shift towards children and young adolescents in a new COVID-19 wave caused by the Delta variant. These modelling results obtained for China are backed up by empirical evidence from other countries, where an upsurge of COVID-19 cases and hospitalizations among children and adolescents has been reported since the summer of 2021 [10,11,44]. Evidence of this epidemiological shift towards children and adolescents also comes from the analyses of local outbreaks in Putian city, China, in September 2021, where 34.1% of the reported cases occurred in those aged 0–11 years [15].

The numbers depicted by the model are partially due to the relatively low effectiveness against Delta variant infection of the vaccines in use in China (51.8%) as of March 2022. Model simulations show that, in the absence of NPIs, even when 93% of the population aged ≥12 years is fully vaccinated, the importation of Delta variant infections could lead to a new COVID-19 wave and substantial health burden. In particular, the estimated number of deaths over six months was 2.4 per 1000 individuals as compared, for instance, with 0.36 deaths per 1000 individuals in the first epidemic wave in Wuhan in early 2020 [3]. Extending vaccination to children could mitigate the number of symptomatic cases and severe outcomes but would not be enough to suppress transmission. Relying on NPIs such as border control screenings, border quarantine, isolation, and mask wearing remains key in this phase of widespread circulation of highly transmissible SARS-CoV-2 variants across the globe. This finding is consistent with studies in France, the UK, and the US where, despite the distribution of more effective vaccines (eg mRNA vaccines and adenoviral vector vaccines), the transmission is far from being suppressed as of November 2021 [47–49].

To return to normal (ie no NPIs), the combination of high vaccine coverage, including children and adolescents, and a highly efficacious vaccine that prevents infection appear to be key to reducing COVID-19 burden to levels closer to those of seasonal influenza. To reach this target, in addition to extending vaccination to children aged 3–11 years, as implemented in China since the end of October 2021, our modelling results suggest that vaccine efficacy against the infection would need to be increased to >88%, slightly higher than the estimated effectiveness of two doses of mRNA-1273 (Moderna) vaccine against Delta variant infection (86.7%, 95% CI: 84.3–88.7%) [50]. China is currently attempting to develop mRNA vaccines [51], vaccines that specifically target the Delta variant [52], multi-valent vaccines, and universal vaccines [53]. However, whether such vaccines will become available in the short term is unknown. Moreover, the waning of vaccine-induced immunity may also worsen the situation [5,48,50]. For this reason, many countries recommended booster shots to population aged ≥18 years as of December 2021 [54]. The debate on whether to prioritize the administration of booster doses to older age groups or extend the vaccination programme to those aged 3–11 years merits further modelling investigations. Currently, the best way to protect infants (aged 0–2 years) appears to be by vaccinating their contacts, such as family members.

Although the inactivated vaccines in use in China as of March 2022 show a relatively low effectiveness against mild and moderate disease caused by Delta variant infection [17,18,20], they appear to be highly effective against severe outcomes [5,18–20]. In the context of a constantly changing virus and the evolving epidemiology of the pandemic, it is of great importance to continue to ramp up efforts to increase vaccination coverage by using currently available vaccines. Promoting equitable access to COVID-19 vaccines is critical to decreasing viral circulation and the likelihood of the emergence of new variants of concern.

We conducted a set of sensitivity analyses to evaluate the impact of the uncertainty surrounding the estimates of COVID-19 epidemiological parameters. These sensitivity analyses show that the main results are essentially unaltered when we assume: (1) homogeneous susceptibility to infection across all age groups, (2) the mixing patterns estimated in Shanghai right after the end of the lockdown in March 2020 [46], (3) a longer generation time, and (4) a lower number of imported infections to seed the epidemic. However, we recognize that, although taken from the literature, parameters regulating COVID-19 burden may differ by country and/or epidemiological context. For instance, the definition of the symptomatic case may be particularly variable across countries and time periods during the pandemic [55,56]. As such, all the obtained results should be interpreted cautiously.

Moreover, our findings should be interpreted by considering the following limitations. First, we assumed that vaccine protection lasted longer than the time of our simulations (six months). However, recent studies have shown that the effectiveness of inactivated vaccines declines to a low level at six months after the second vaccine dose [5]. Second, we estimated the disease burden potentially caused by the importation of Delta variant infections in the absence or presence of NPIs. The impact of NPIs has been modelled through a simple reduction in transmissibility occurring homogenously with age. We still lack evidence regarding whether this is the case or whether NPIs lead to age-related effects. Moreover, our analysis is not suited to pinpoint which NPIs need to be performed to reach the considered levels of transmission reduction. Further studies exploring this direction are required.

This study focuses on the estimation of COVID-19 burden and its potential age-shift towards younger ages in case of a widespread epidemic caused by the Delta variant in mainland China. As of March 2022, although no longer dominant, Delta is still circulating in countries neighbouring China and have caused local outbreaks in China – see for instance the outbreak in Hohhot, Inner Mongolia that started on 15 February 2022 and caused more than 450 cases [57]. Together with the increased severity of Delta as compared to Omicron [58], this highlights the need to assess the potential outcome of a nationwide epidemic of Delta in mainland China. Nonetheless, we acknowledge the need to perform a similar assessment for the Omicron variant. The higher transmissibility and increased immune evasive capability of the Omicron variant compared to the Delta variant suggest that the virus could reach any segment of the population, especially those with lower vaccination coverage (ie children). To provide a systematic assessment of COVID-19 burden, specific estimates of efficacy against Omicron infection and subsequent clinical endpoints for the vaccines administered in China would be needed as well as estimates of the association between Omicron infection and clinical progression. Such an investigation deserves a further investigation.

In sum, although vaccination is key to dramatically reducing severe outcomes of SARS-CoV-2 infections and the overall burden of the COVID-19 pandemic, in the absence of NPIs, the importation of Delta variant infections could lead to widespread transmission and substantial disease burden in mainland China, even with vaccination coverage as high as 89% of those aged ≥12 years (VE = 51.8% in preventing the infection). Moreover, vaccination programmes targeting individuals aged ≥12 years only can potentially shift the COVID-19 burden towards younger ages. Extending vaccination to children aged 3–11 years would mitigate the disease burden within this age group, but would not be enough to suppress transmission. Our findings highlight the importance of maintaining a certain degree of NPIs (eg mask-wearing, enhanced testing, social distancing, reducing mass gatherings) to prevent excessive COVID-19 burden in the case of a nationwide epidemic. Moreover, our results show the need to strengthen vaccination efforts by simultaneously extending the target age groups of the vaccination campaign and increasing vaccine protection via booster vaccination or through the administration of more efficacious vaccines. In particular, the development of new highly efficacious vaccines should be a point of emphasis for China as well as for the rest of the world.

## Supplementary Material

Supplemental Material

## Data Availability

The data and code that support the findings of this study are available on GitHub at https://github.com/caijun/Delta_burden.
