# Supplementary material for: Assessing the transition of COVID-19 burden towards the young population while vaccines are rolled out in China*
Source: Emerg Microbes Infect. 2022 Apr 25;11(1):1205–14. doi: 10.1080/22221751.2022.2063073 (PMC9045766; doi:10.1080/22221751.2022.2063073)
Supplement: Supplemental Material [file TEMI_A_2063073_SM3268.docx]

Supplementary Information for:

**Assessing the transition of COVID-19 burden towards the young population while vaccines are rolled out in China**

Jun Cai^1^*, Juan Yang^1,2,3^*, Xiaowei Deng^1^, Cheng Peng^1^, Xinhua Chen^1^, Qianhui Wu^1^, Hengcong Liu^1^, Juanjuan Zhang^1,2,3^, Wen Zheng^1^, Junyi Zou^1^, Zeyao Zhao^1^, Marco Ajelli^4†^, Hongjie Yu^1,2,3†^

1. Department of Infectious Diseases, Huashan Hospital, School of Public Health, Fudan University
2. Key Laboratory of Public Health Safety, Ministry of Education, Shanghai, China
3. Shanghai Institute of Infectious Disease and Biosecurity, Fudan University, Shanghai, China
4. Laboratory for Computational Epidemiology and Public Health, Department of Epidemiology and Biostatistics, Indiana University School of Public Health, Bloomington, IN, USA

*These authors contributed equally to this work.

^†^These authors are joint senior authors contributed equally to this work.

Corresponding authors: Hongjie Yu, Shanghai Institute of Infectious Disease and Biosecurity, School of Public Health, Fudan University, Shanghai 200032, China

E-mail: [yhj@fudan.edu.cn](mailto:yhj@fudan.edu.cn)

Contents

[**1.** **Materials and methods** 3](#_Toc98534653)

[**1.1.** **SARS-CoV-2 transmission and vaccination model** 3](#_Toc98534654)

[**1.2.** **Daily vaccination capacity** 8](#_Toc98534655)

[**1.3.** **COVID-19 burden model** 9](#_Toc98534656)

[**1.4.** **Vaccine effectiveness** 11](#_Toc98534657)

[**Table S1. Summary of key parameters used in the model and sensitivity analyses** 14](#_Toc98534658)

[**Table S2. Overall population, proportions of those with contraindications to vaccination, and pregnant women by age group in China** 15](#_Toc98534659)

[**2.** **Sensitivity analyses** 16](#_Toc98534660)

[**2.1.** **Homogeneous susceptibility to infection by age** 16](#_Toc98534661)

[**2.2.** **Contact patterns in the post-lockdown period** 17](#_Toc98534662)

[**2.3.** **Number of initial seed infectors** 18](#_Toc98534663)

[**2.4.** **Generation time** 19](#_Toc98534664)

[**3.** **Additional results** 21](#_Toc98534665)

[**References** 23](#_Toc98534666)

1. **Materials and methods**
   1. **SARS-CoV-2 transmission and vaccination model**

We developed an age-structured stochastic susceptible-infectious-removed (SIR) model to simulate SARS-CoV-2 transmission and vaccination. The Chinese population was divided into 16 age groups, as presented in Table S2. We considered a baseline scenario, where:

- We used age-mixing patterns specific to China that were quantified during the pre-pandemic period^1^.
- We assumed an age-dependent susceptibility to SARS-CoV-2 Delta infection that was lower in children under 15 years and higher in adults aged 65+ years (Table S1)^2^.
- Asymptomatic and symptomatic individuals are assumed to be equally infectious, and infectiousness is assumed to be the same across age groups^2^.
- A two-dose vaccination is assumed to reduce an individual’s susceptibility to SAR-CoV-2 infection. We consider the vaccine effectiveness (VE) against SARS-CoV-2 Delta infection to be homogeneous across age groups^3-5^.
- As vaccine breakthrough cases increase^6,7^, we consider a “leaky” vaccine where all vaccinated individuals are exposed to a lower risk of infection, which is 1-VE times that of non-vaccinated individuals^8^.
- We assumed that vaccine- or natural infection-induced protection lasts longer than the period considered (6 months)^9^.

To test the impact of age-mixing patterns on the transmission of imported Delta variant infections, a sensitivity analysis considering an alternative contact matrix for China estimated in March 2020 (post-lockdown period)^10^ was explored. In addition, homogeneous susceptibility to infection across all age groups was explored as a sensitivity analysis (see Sec. 2.1).


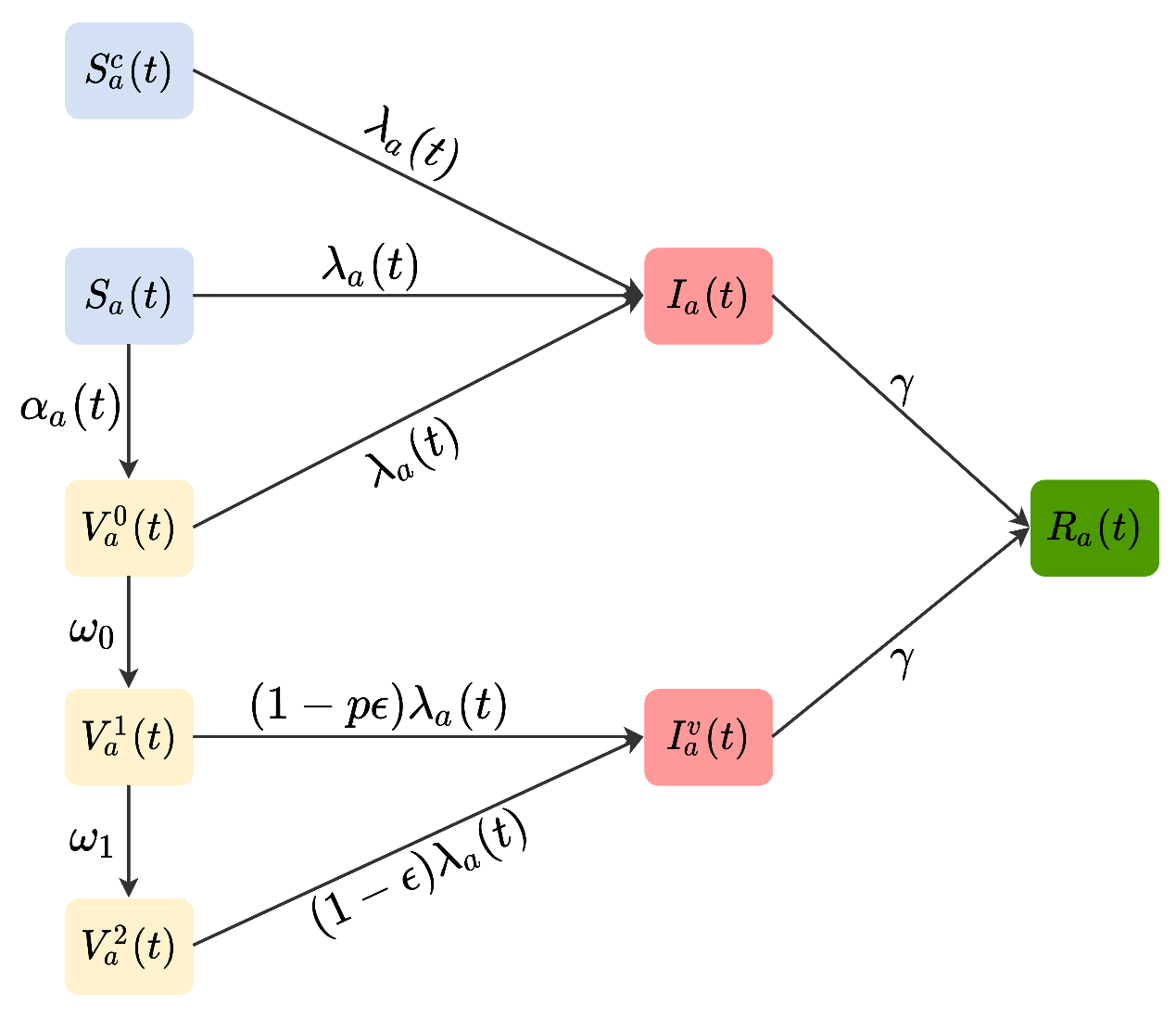


**Figure S1. Schematic representation of SARS-CoV-2 transmission and vaccination model.** Taking the age group $a \in[1, 16]$ at time $t$ as an example, model compartments are defined as follows: unvaccinated susceptible individuals who are ineligible for vaccination due to contraindications and pregnancy $S_{a}^{c}\left( t \right)$, unvaccinated susceptible individuals who are eligible for vaccination $S_{a}(t)$, unvaccinated infected individuals $I_{a}(t)$, removed individuals $R_{a}(t)$, unprotected individuals who received their first dose within 21 days before time *t*$V_{a}^{0}\left( t \right)$, partially vaccinated individuals who received their second dose within 14 days before time *t* $V_{a}^{1}\left( t \right)$, vaccinated individuals who received their second dose at least 14 days before time *t* $V_{a}^{2}\left( t \right)$, and vaccinated infected individuals $I_{a}^{v}(t)$. Note that vaccinated individuals are defined as those administered two doses. The model parameters include: time- and age-dependent force of infection $\lambda_{a}(t$), probability of administration of the first dose ($\alpha_{a}\left（ t \right）$), recovery rate from infection $\gamma$, relative vaccine efficacy against infection right after administration of the second dose *p* as compared with maximum protection (i.e., after ramp-up of the second dose) $\epsilon$, time interval between the administration of the first and second dose ($1/{\omega_{0})}$and maximum protection ($1/{\omega_{1}}$).

The SARS-CoV-2 transmission and vaccination model is schematically represented in Figure S1 and described by the following system of differential equations:

$$\frac{dS_{a}^{c}\left( t \right)}{dt}=-\lambda_{a}\left( t \right)S_{a}^{c}\left( t \right),$$

$$\frac{dS_{a}\left( t \right)}{dt}=-\lambda_{a}\left( t \right)S_{a}\left( t \right)-\alpha_{a}\left( t \right)S_{a}\left( t \right),$$

$$\frac{dI_{a}\left( t \right)}{dt}=\lambda_{a}\left( t \right)\left( S_{a}^{c}\left( t \right)+S_{a}\left( t \right)+V_{a}^{0}\left( t \right) \right)-\gamma I_{a}\left( t \right),$$

$$\begin{aligned} \frac{{dV}_{a}^{0}\left( t \right)}{dt}=\alpha_{a}\left( t \right)S_{a}\left( t \right)-\lambda_{a}\left( t \right)V_{a}^{0}\left( t \right)-\omega_{0}V_{a}^{0}\left( t \right),\#\left( 1 \right) \end{aligned}$$

$$\frac{{dV}_{a}^{1}\left( t \right)}{dt}=\omega_{0}V_{a}^{0}\left( t \right)-\left( 1-p\epsilon\right)\lambda_{a}\left( t \right)V_{a}^{1}\left( t \right)-\omega_{1}V_{a}^{1}\left( t \right),$$

$$\frac{dV_{a}^{2}\left( t \right)}{dt}=\omega_{1}V_{a}^{1}\left( t \right)-\left( 1-\epsilon\right)\lambda_{a}\left( t \right)V_{a}^{2}\left( t \right),$$

$$\frac{dI_{a}^{v}\left( t \right)}{dt}=\left( 1-p\epsilon\right)\lambda_{a}\left( t \right)V_{a}^{1}\left( t \right)+\left( 1-\epsilon\right)\lambda_{a}\left( t \right)V_{a}^{2}\left( t \right)-\gamma I_{a}^{v}\left( t \right),$$

$$\frac{dR_{a}\left( t \right)}{dt}=\gamma\left( {I_{a}\left( t \right)+I}_{a}^{v}\left( t \right) \right),$$

where:

- $S_{a}^{c}$ represents the number of unvaccinated susceptible individuals who are ineligible for vaccination due to contraindications and pregnancy in age group *a* (Table S2).
- $S_{a}$ represents the number of unvaccinated susceptible individuals who are eligible for vaccination in age group *a* (Table S2).
- $I_{a}$ represents the number of unvaccinated infected individuals of age group *a*.
- $R_{a}$ represents the number of recovered or removed individuals of age group *a*.
- $V_{a}^{0}$ represents the number of individuals administered the first dose in age group *a*. We assumed that the second dose was administered 21 days after the first dose, namely $1/{\omega_{0}}=21$ days.
- $V_{a}^{1}$ represents the number of individuals in age group *a* who were administered their second dose within 14 days. The interval between administration and full protection of the second dose was 14 days, namely $1/{\omega_{1}}=14$ days.
- $V_{a}^{2}$ represents the number of individuals of age group *a* who received their second dose for at least 14 days.
- $I_{a}^{v}$ represents the number of vaccinated infected individuals of age group *a*.
- $\epsilon$ represents the expected VE against infection after ramp-up of the second dose.
- *p* represents the relative VE against infection right after the administration of the second dose as compared with the maximum protection $\epsilon$.

Susceptible individuals of age group *a* at time *t* are exposed to a time- and age-dependent force of infection $\lambda_{a}(t)$, which is defined as:

$$\begin{aligned} \lambda_{a}\left( t \right)=(1-\varphi)\beta r_{a}\sum_{\tilde{a}} C_{a, \tilde{a}}\frac{I_{\tilde{a}}+I_{\tilde{a}}^{v}}{N_{\tilde{a}}},\#\left( 2 \right) \end{aligned}$$

where:

- $\beta$ is a scaling factor shaping SARS-CoV-2 transmissibility in the absence of non-pharmaceutical interventions (NPIs) such as face masks or hand hygiene precautions, computed by assuming basic reproduction number *R_0_* = 6^11^, as estimated for the SARS-CoV-2 Delta variant.
- $\varphi$ is a coefficient representing the reduction in transmissibility due to NPIs.
- $r_{a}$ is the relative susceptibility to SARS-CoV-2 infection in age group *a* (Table S1).
- $C_{a,\tilde{a}}$ represents the age-group-specific contact matrix, whose elements describe the mean number of daily contacts that an individual in age group *a* has with individuals in age group $\tilde{a}$ (Figure S4).
- $N_{\tilde{a}}$ represents the number of individuals in age group $\tilde{a}$ (Table S2).

Given the value of *R_0_* = 6, the distribution of the age-specific susceptibility profile (*r_a_*), and the distribution of the bootstrapped contact matrix $C_{a,\tilde{a}}$, the distribution of transmission rate $\beta$ is calculated analytically through the equation described in our previous work^12^. When considering a set of NPIs that are capable of bringing the net reproduction number to a value *R_e_* < *R_0_*, we rescale the transmission rate $\beta$ by a factor ($1-\varphi$), where $\varphi=1-R_{e}/R_{0}$. We tested a range value of *R_e_* = 1.1~5.9 with a step of 0.2 to explore the impact of different intensities of NPIs adopted on the disease burden from Delta variant infections in China.

Given that vaccinated and unvaccinated individuals infected with the Delta variant have similar proliferation and peak Ct values^13-15^, it appears that there is a limited difference in infectiousness despite vaccination. Thus, we assumed that all infectious compartments despite vaccination status ($I_{a}$ and $I_{a}^{v}$) have the same average duration of infectiousness ($1/\gamma$), which corresponds to the length of generation time in an SIR model^16^. As studies conducted in UK and in China show that the Delta variant transmits more quickly than previously circulating variants^17,18^, we considered an average generation time of 4.6 days^18^ in the main analysis. An analysis conducted in Singapore found no significant difference in serial intervals between the Delta variant and the original lineages^19^. Therefore, in a sensitivity analysis, we considered a generation time of 7 days, in line with estimates for the original lineages^20^ (see Sec. 2.4).

We simulated the daily vaccination process in China as follows. First, we prioritised those individuals who need to receive their second dose at time *t*; then, the remaining doses are randomly administered to those who are eligible to receive their first dose. At each time *t*, the first dose is administered to a fraction $\alpha_{a}(t)$ of unvaccinated susceptible individuals in age group *a*:

$$\begin{aligned} \alpha_{a}\left( t \right)=\frac{d_{a}\left( t \right)}{S_{a}\left( t \right)},\#\left( 3 \right) \end{aligned}$$

where $d_{a}(t)$ represents the number of first doses to be administered to individuals in age group *a* at time *t* under the designed vaccination strategies.

We assumed that the first dose does not confer protection^21,22^, that partially vaccinated individuals who were administered their second dose within 14 days are protected by a proportion *p* of the expected vaccine efficacy $\epsilon$ (i.e., $p\epsilon$), and that fully vaccinated individuals who were administered their second dose after at least 14 days are protected by the expected vaccine efficacy $\epsilon$ (see Sec. 1.4). The proportion *p* = 0.84 (42.5%/50.7%) was estimated from the efficacies of CoronaVac vaccine reported in the PROFISCOV study^4^. The CoronaVac vaccine is one of inactivated vaccines widely used in the mass vaccination program in China.

Simulation results were obtained using a stochastic version of the model described above with a time step of 0.25 days. A total of 200 simulations were run for each scenario, sampling at each simulation a different value from the joint distribution of transmission rate $\beta$, the bootstrapped contact matrices $C_{a,\tilde{a}}$, and the age-specific susceptibility profile *r_a_*.

- 1. **Daily vaccination capacity**

As described in our previous work^11^, the daily vaccination capacity is based on the cumulative number of doses of COVID-19 vaccines administered in China released by the National Health Commission (NHC) until February 1, 2022^23^. The first data on cumulative doses administered in China was released on November 30, 2020. Until March 22, 2021, the NHC began to regularly report the cumulative number of doses administered in China per day. Before March 22, the daily vaccination capacity was interpolated by assuming a constant daily vaccination rate between two adjacent available data points.

Based on the cumulative doses (3.53 millions) allocated to children aged 3-11 years as of October 29, 2021^24^ and the doses (3.332 millions) administered at October 28^25^, 2021, we assumed that the roll-out started to include children aged 3-11 years at October 28, 2021. As the daily vaccination capacity reported between October 28, 2021 and February 1, 2022 also included booster doses, to obtain the daily doses administered to children aged 3-11 years during the same period, we first obtained the number of daily booster doses administered in China via interpolation and then subtracted them from the daily vaccination capacity. The NHC occasionally released the cumulative number of booster doses administered in China, which were interpolated by assuming a constant daily booster administration rate between two adjacent available data points. The daily doses administrated to children aged 3-11 years are shown in Figure S2.


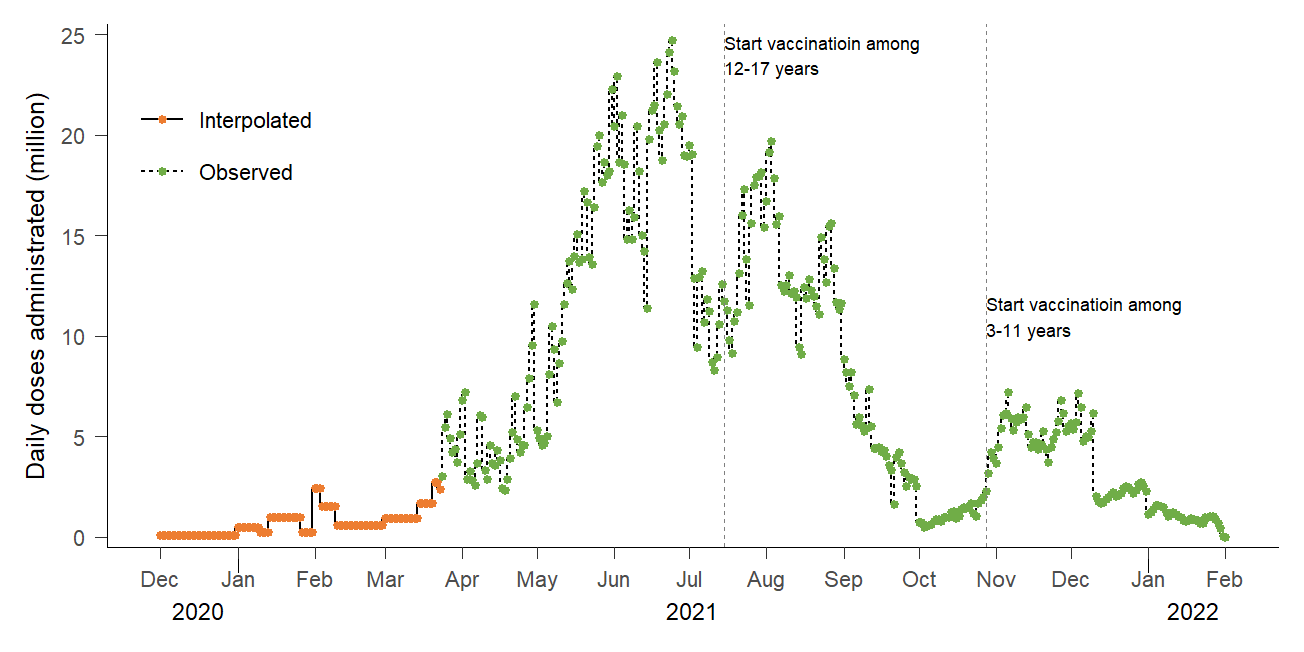


**Figure S2. Daily number of vaccine doses administered in China.** The daily doses administered in China between March 22, 2021 and February 1, 2022, were obtained from the National Health Commission. Before March 22, 2021, the daily doses administered were interpolated. Note that the curve after October 28, 2021 represents the daily doses administered to children aged 3-11 years.

- 1. **COVID-19 burden model**

The main model outputs are the age-specific number of new infections per day in unvaccinated and vaccinated individuals $i_{a}(t)$ and $i_{a}^{v}\left( t \right), t=1, 2, \cdots, 182$ (simulation period from December 1, 2021 to June 1, 2022). Mathematically, these are computed as follows:

$$\begin{aligned} i_{a}\left( t \right)=I_{a}\left( t+1 \right)-\left( 1-\gamma\right)I_{a}\left( t \right), \#\left( 4 \right) \end{aligned}$$

$$\begin{aligned} i_{a}^{v}\left( t \right)=I_{a}^{v}\left( t+1 \right)-\left( 1-\gamma\right)I_{a}^{v}\left( t \right). \#\left( 5 \right) \end{aligned}$$

The COVID-19 burden of each vaccination scenario was evaluated in terms of the cumulative incidence of symptomatic cases, hospitalisations, ICU admissions, and deaths over the 6 months $B^{type}$, $type\in\{symp, hosp, icu, death\}$, with:

$$\begin{aligned} B^{type}=\sum_{t=1}^{182} \sum_{a=1}^{16} \left( {r_{a}^{type}i}_{a}\left( t \right)+r_{a}^{v\_type}i_{a}^{v}\left( t \right) \right), \#\left( 6 \right) \end{aligned}$$

where $r_{a}^{type}$ represents the risks of developing symptoms, being hospitalized, being admitted to ICUs, and dying among unvaccinated persons infected the Delta variant in age group *a*; $r_{a}^{v\_type}$ represents the corresponding age-specific risks among vaccinated persons infected with the Delta variant. $r_{a}^{type}$ and $r_{a}^{v\_type}$ are estimated in following equations (7-9). Note that we are interested in the total incidence of these epidemiological outcomes. Therefore, we did not account for delays between infection and different clinical outcomes (i.e., symptomatic cases, hospitalisations, ICU admissions, and deaths).

Scarce information on the risks of progressing from infection to different clinical outcomes for the Delta variant has been reported in China because dozens of local outbreaks caused by imported Delta infections were quickly contained. Meanwhile, an increasing number of studies suggest that the Delta variant is associated with a greater risk of adverse outcomes, including hospitalisation, ICU admission, and death for patients, than the wild-type or Alpha variants^26-29^. Therefore, we estimated the age-specific risk $r_{a}^{type}$, $type\in\{symp, hosp, icu, death\}$ using the following equation:

$$\begin{aligned} r_{a}^{type}=r_{a, WT}^{type}\times\Delta_{WT\to Delta}^{type}, \#\left( 7 \right) \end{aligned}$$

where $r_{a, WT}^{type}$ represents the risks of developing symptoms, being hospitalized, being admitted to ICUs, and dying among those infected with the wild-type strain in age group *a*. $\Delta_{WT\to Delta}^{type}$ represents the (adjusted) risk ratio (or hazard ratio) of the corresponding clinical outcomes caused by the Delta variant compared with the wild-type variant.

The age-specific risks $r_{a, WT}^{type}$ for the wild-type infection are presented in Table 1, which were estimated as described in our previous work^12^. By pooling different studies from China^26^, Scotland^27^, Canada^29^ and England^28,30^, we estimated the risk ratio associated with the Delta variant as $\Delta_{WT\to Delta}^{hosp}$= 2.78 (1.92–4.13) for hospitalisation, $\Delta_{WT\to Delta}^{icu}$= 3.17 (1.95–5.59) for ICU admission, and $\Delta_{WT\to Delta}^{death}$= 2.33 (1.54–3.31) for death compared to the wild-type strain, respectively. However, we assumed $\Delta_{WT\to Delta}^{symp}$= 1, inspired by the finding that there was no significant change in the symptoms reported by those with the Alpha or wild-type variants^31^ (Table 1). We estimated $\Delta_{WT\to Delta}^{hosp}$ by linking the risk ratio of hospitalisation associated with the Delta variant compared to the Alpha variant, and the risk ratio of hospitalisation associated with the Alpha variant compared to the wild-type strain.

Reduced risks of clinical outcomes are observed for vaccinated infections compared to unvaccinated infections^32^. We adopted below method^33^ to adjust the risks of progressing from infection to different clinical outcomes for vaccinated individuals, $r_{a}^{v\_type}$, with:

$$\begin{aligned} r_{a}^{v\_type}=r_{a}^{type}\times\left( 1-\epsilon^{type|infect} \right), \#\left( 8 \right) \end{aligned}$$

$$\begin{aligned} \epsilon^{type}=\epsilon+\left( 1-\epsilon\right)\times\epsilon^{type|infect}, \#\left( 9 \right) \end{aligned}$$

where $\epsilon^{type|infect}$ represents the VE in preventing different clinical outcomes conditionally on infection with the Delta variant; while $\epsilon^{type}$ represents the overall VE in preventing symptomatic infection, hospitalisation, ICU admission, and death (Table 1; see Sec. 1.4 for details).

- 1. **Vaccine effectiveness**

The overall effectiveness of inactivated COVID-19 vaccines against different clinical endpoints (i.e., infection, symptomatic cases, hospitalisation, ICU admission, and death) caused by the Delta variant are used in our model. We obtained effectiveness of full vaccination against infection caused by the Delta variant ($\epsilon$=51.8%, 95% CI: 20.3–83.2%) from a cohort study on the outbreak of Delta variant in Guangdong, China^22^. The effectiveness of full vaccination against symptomatic COVID-19 caused by the Delta variant ($\epsilon^{symp}$=60.4%, 95% CI: 31.8–88.9%) is also obtained from the same cohort study. Other real-world studies^21,34^ conducted in China reported similar VEs against symptomatic disease caused by the Delta variant.

A real-world study in Brazil^35^ reported the VEs of inactivated CoronaVac against hospitalisation or death caused by the Delta variant. Although the dominant variants detected in Brazil during the study period (February 24, 2020 to November 11, 2021) were Gamma and Delta, we assumed that the inactivated vaccines used in China have similar VEs in preventing hospitalisation or death associated with the Delta variant (i.e., $\epsilon^{hosp}$=82.6%, 95% CI: 82.0–83.1%; $\epsilon^{death}$=83.6%, 95% CI: 82.8–84.3%). A similar VE of two doses of inactivated COVID-19 vaccines against severe illness caused by the Delta variant (82%) was also reported in a real-world study conducted in China^34^. We further assumed that the effectiveness of inactivated COVID-19 vaccines against ICU admission caused by the Delta variant is the same as that against death (i.e., $\epsilon^{icu}=\epsilon^{death}$).

Considering that the VE against the Delta variant infections might be improved in the future (e.g., boosting with a third dose), we explored a range value of $\hat{\epsilon}$=50%~95% with a step of 5%. When calculating the COVID-19 burden of these scenarios, the VE against different clinical endpoints, $\hat{\epsilon}^{type}$, $type\in\{symp, hosp, icu, death\}$, were adjusted using the following equation:

$$\begin{aligned} \hat{\epsilon}^{type}=\max\left( {k\times\epsilon}^{type},\epsilon_{0}^{type} \right)\#\left( 10 \right) \end{aligned}$$

where $k=\hat{\epsilon}/\epsilon$, represents the fold change in VE against infection. $\epsilon_{0}^{symp}$ = 96.4%, represents the reported highest VE against symptomatic COVID-19 caused by SARS-CoV-2 variants^36^, whereas $\epsilon_{0}^{hosp}=\epsilon_{0}^{icu}=\epsilon_{0}^{death}$ = 100%, respectively represents the reported highest VE against hospitalisation, ICU admission, and death.

A summary of the model parameters is presented in Table 1 in the main text, as well as in Tables S1 and S2.

**Table S1. Summary of key parameters used in the model and sensitivity analyses**

| **Parameter** | **Description** | **Value (or range)** | **Sensitivity analysis** |
| --- | --- | --- | --- |
| **Demographic** | | | |
| *N_a_* | Population size for age group *a* | See Table S2 ^37^ | - |
| $C_{a,\tilde{a}}$ | Age-group-specific contact matrix | Contact matrix in Shanghai before the pandemic (Figure S4A) ^1^ | Contact matrix for Shanghai in post-lockdown period (Figure S4B) ^10^ |
| **Epidemiology** | | | |
| $1/\gamma$ | Generation time (days) | 4.6 ^18^ | 7 ^20^ |
| *r_a_* | Relative susceptibility to SARS-CoV-2 infection at age *a* | $r_{a}=0.58$ (95% CI 0.34–0.98) when $a<15$;  $r_{a}=1$ for $15\leq a<65$; $r_{a}=1.65$ (95% CI 1.03–2.65) when $a\geq65$ ^2^ | $r_{a}=1$ for all age groups (homogeneous susceptibility) |
| *n_0_* | Initial seed infectors | 40 ^12^ | 10, 20 |
| **Vaccination** | | | |
| $1/\omega_{0}$ | Interval between the administration of the first dose and second dose (days) | 21 ^3^ | - |
| $1/\omega_{1}$ | Delay between the administration of the second dose and achievement of the expected vaccine efficacy (days) | 14 ^3^ | - |
| *p* | Relative vaccine efficacy between 0 and $1/\omega_{1}$ days after the administration of the second dose compared to the expected vaccine efficacy | 0.84 ^4^ | - |

**Table S2. Overall population, proportions of those with contraindications to vaccination, and pregnant women by age group in China**

| **ID** | **Age group** | **Population** | **Contraindications (%)** | **Pregnant women (%)** |
| --- | --- | --- | --- | --- |
| 1 | 0–2 | 47,208,873 | 0.2 | 0.0 |
| 2 | 3–11 | 158,390,745 | 0.1 | 0.0 |
| 3 | 12–17 | 98,365,257 | 0.1 | 0.3 |
| 4 | 18–24 | 120,465,522 | 0.2 | 4.0 |
| 5 | 25–29 | 97,989,003 | 0.2 | 10.3 |
| 6 | 30–34 | 128,738,970 | 0.3 | 5.2 |
| 7 | 35–39 | 100,091,455 | 0.5 | 2.6 |
| 8 | 40–44 | 96,274,146 | 0.7 | 0.7 |
| 9 | 45–49 | 119,837,617 | 0.9 | 0.3 |
| 10 | 50–54 | 123,445,382 | 1.2 | 0.0 |
| 11 | 55–59 | 98,740,491 | 1.7 | 0.0 |
| 12 | 60–64 | 77,514,139 | 2.2 | 0.0 |
| 13 | 65–69 | 74,149,766 | 2.7 | 0.0 |
| 14 | 70–74 | 44,949,689 | 3.2 | 0.0 |
| 15 | 75–79 | 26,544,616 | 3.2 | 0.0 |
| 16 | ≥80 | 26,618,103 | 2.6 | 0.0 |

1. **Sensitivity analyses**
   1. **Homogeneous susceptibility to infection by age**

We evaluated the model sensitivity to the assumption that susceptibility to infection is reduced in children and increased in older adults. In fact, while this may have been the case for historical lineages^2,38^, it may not necessarily be correct for the Delta variant. Under an alternative assumption of an equal susceptibility to infection across all age groups, we recalibrated the value of $\beta$ and applied all other conditions as in the baseline scenario. We estimated that the disease burden decreased slightly, ranging from 2% to 8% for symptomatic cases, hospitalisations and ICU admissions, and about 12% for death as compared to the baseline, for the "adults+adolescents+children" vaccination strategy (Figure S3).

**
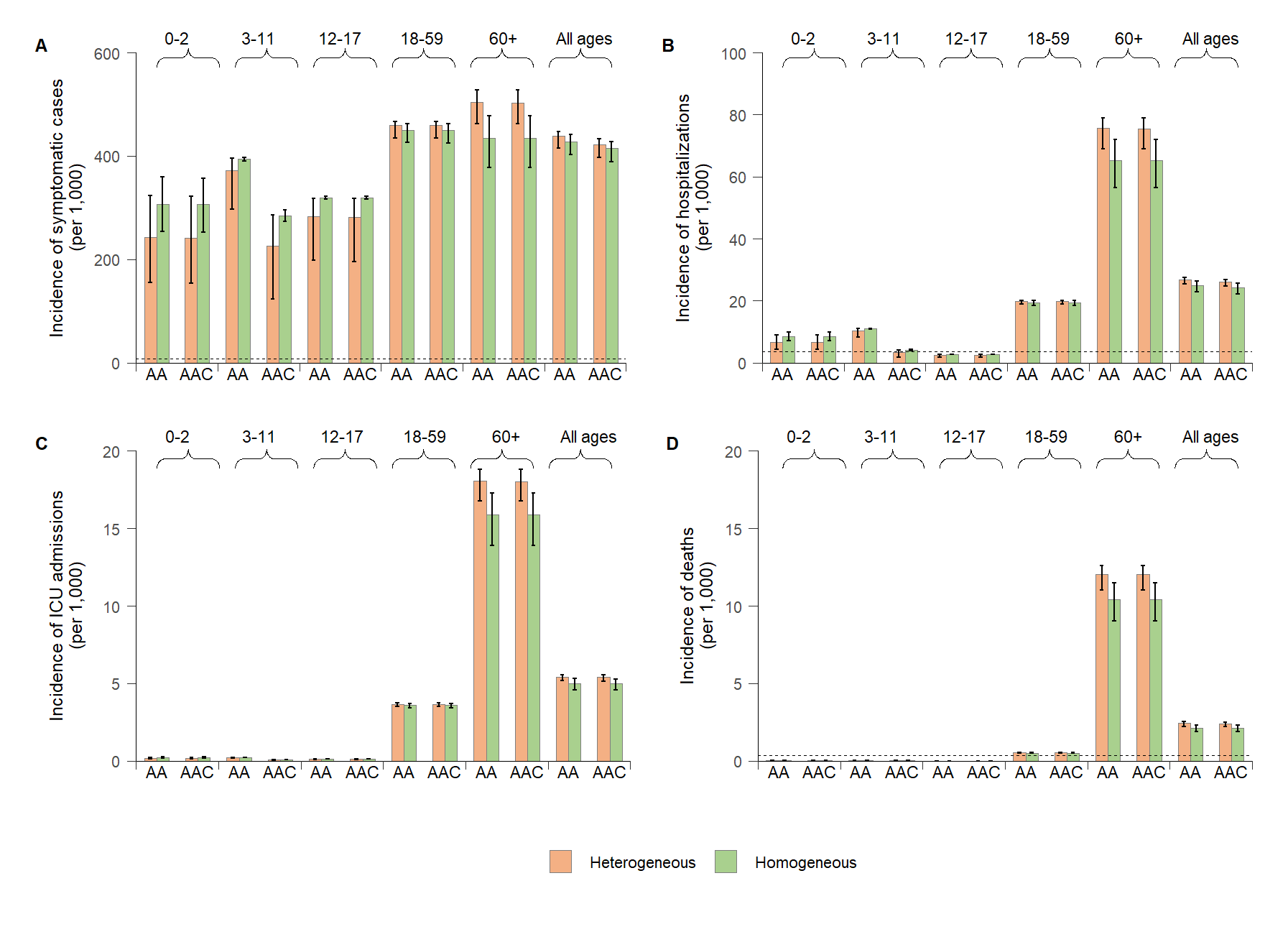
**

**Figure S3. Disease burden due to SARS-CoV-2 Delta variant infections in China assuming homogeneous or heterogeneous susceptibility to infection. A** Cumulative number of symptomatic cases per 1,000 individuals after 6 simulated months by vaccination strategy (AA = "adults+adolescents" vaccination strategy, AAC = "adults+adolescents+children" vaccination strategy) and age group for heterogeneous (baseline) and homogeneous susceptibility to infection by age. **B** As **A**, but for the incidence of hospitalisations. **C** As **A**, but for the incidence of ICU admissions. **D** As **A**, but for the incidence of deaths. The horizontal dotted lines in **A**, **B**, and **D** respectively represent the rates of symptomatic cases, hospitalisations, and deaths of the first pandemic wave of COVID-19 in Wuhan, China^39^.

- 1. **Contact patterns in the post-lockdown period**

We adjusted the age groups of the contact matrix according to the age groups classified in the mass vaccination campaign in China. Age-mixing patterns specific to Shanghai, China in the pre-pandemic period were used in the main analysis^1^ (Figure S4A). As the physical distancing policies adopted during the pandemic have changed the population contact patterns^10,40^, we evaluated the model sensitivity to contact patterns derived from different periods. Using an alternative age-mixing pattern in Shanghai, China, estimated in March 2020 (post-lockdown period)^10^ (Figure S4B), we recalibrated the value of $\beta$ and applied all other conditions as in the baseline scenario. We estimated a lower than 1% variation in disease burden in terms of symptomatic cases, hospitalisations, ICU admissions, and deaths, compared to the baseline under the "adults+adolescents+children" vaccination strategy (Figure S5).


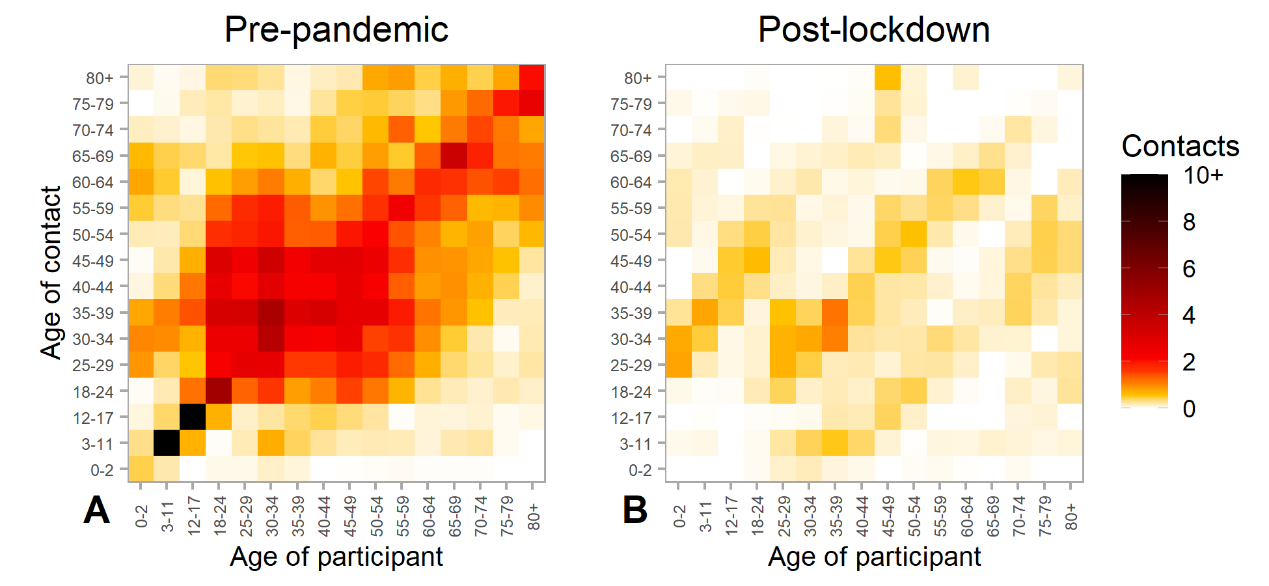


**Figure S4. Age-mixing patterns in China. A** Pre-pandemic contact matrix, which is used in the main analysis. Mixing patterns refer to Shanghai, China in 2017/2018^1^. Each cell of the matrix represents the mean number of daily contacts that an individual in a given age group has with other individuals, stratified by age group. The colour intensity represents the number of daily contacts. **B** As **A**, but for the post-lockdown contact matrix. Mixing patterns refer to Shanghai, China, in March 2020, when interventions were relaxed after the lockdown^10^.

**
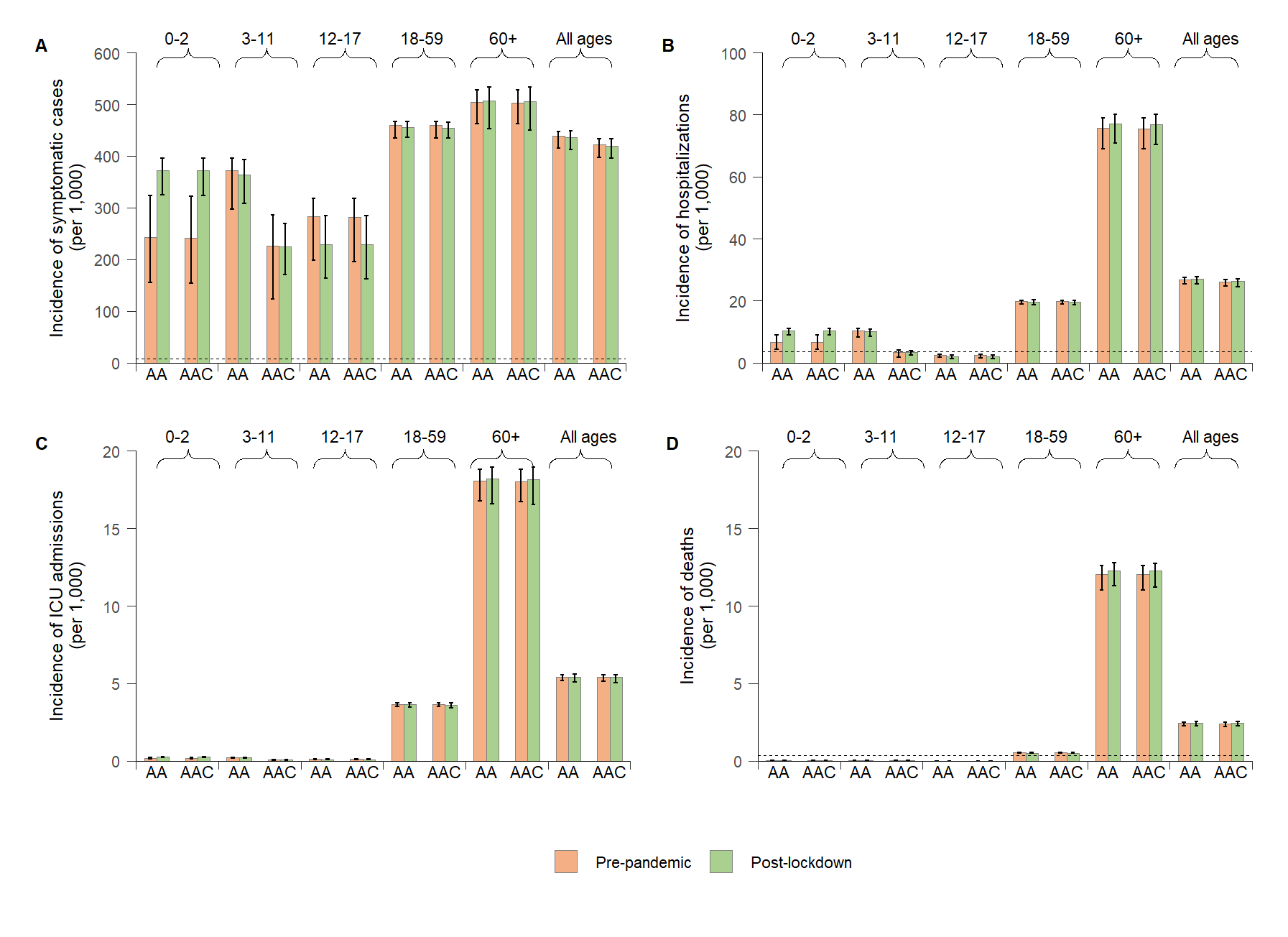
**

**Figure S5. Disease burden due to SARS-CoV-2 Delta variant infections in China using age-mixing patterns estimated in the pre-pandemic or post-lockdown period. A** Cumulative number of symptomatic cases per 1,000 individuals after 6 simulated months by vaccination strategy (AA = "adults+adolescents" vaccination strategy, AAC = "adults+adolescents+children" vaccination strategy) and age group for pre-pandemic (baseline) and post-lockdown contact patterns. **B** As **A**, but for the incidence of hospitalisations. **C** As **A**, but for the incidence of ICU admissions. **D** As **A**, but for the incidence of deaths. The horizontal dotted lines in **A**, **B**, and **D** respectively represent the rates of symptomatic cases, hospitalisations, and deaths of the first pandemic wave of COVID-19 in Wuhan, China^39^.

- 1. **Number of initial seed infectors**

In the main analysis, 40 imported infections are seeded to trigger an epidemic. Under China's dynamic “zero-COVID” policy, it’s more likely a small number of imported infections that would trigger local outbreaks. Thus, we performed a sensitivity analysis on the number of initial seed infectors and repeated the baseline simulations with 10 and 20 seeding imported infections (Table S1). The resulting disease burden are robust to the number of initial seed infectors across vaccination strategies and age groups (Figure S6).

**
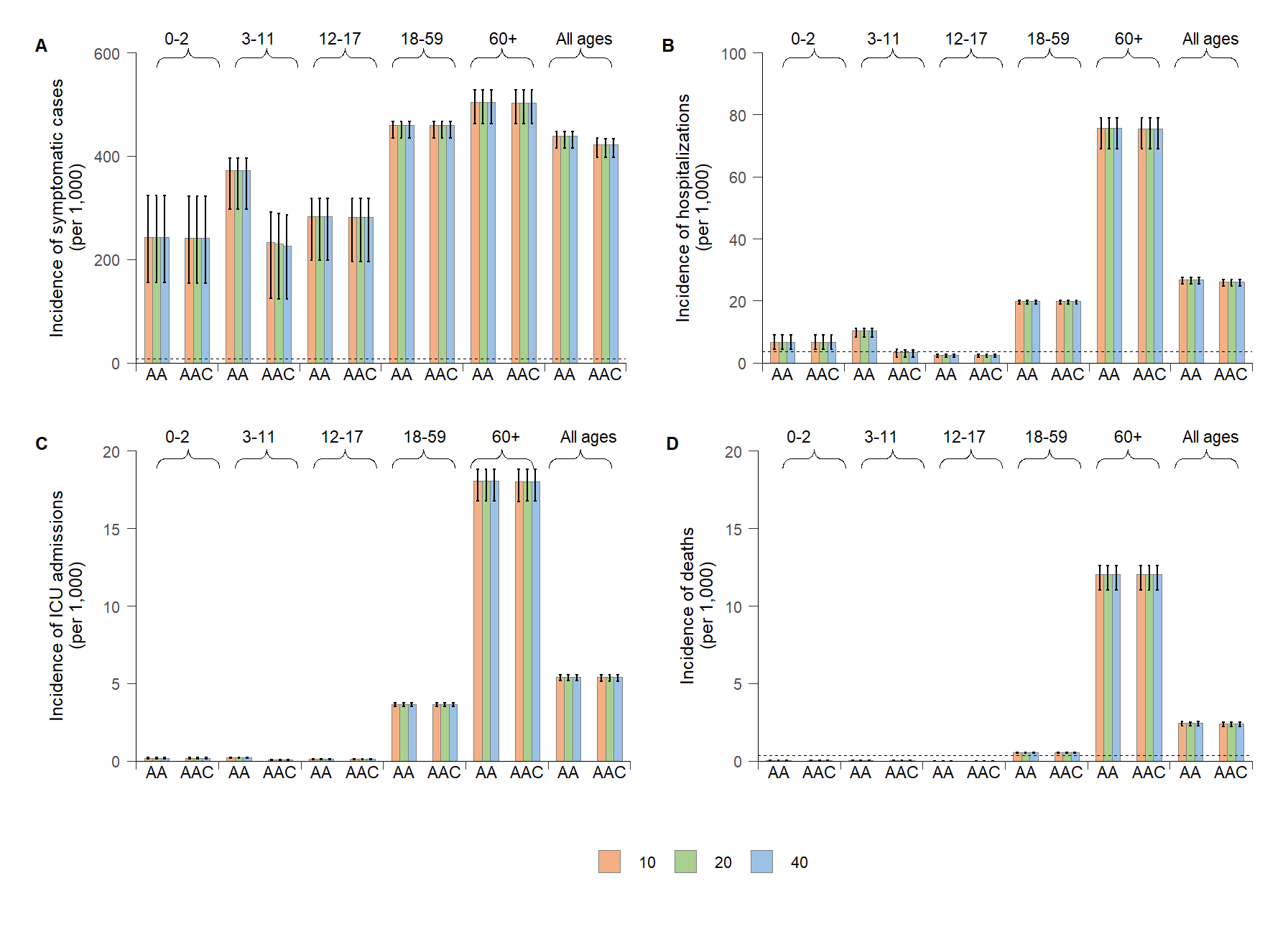
**

**Figure S6. Disease burden due to SARS-CoV-2 Delta variant infections in China assuming varying number of initial seed infectors. A** Cumulative number of symptomatic cases per 1,000 individuals after 6 simulated months by vaccination strategy (AA = "adults+adolescents" vaccination strategy, AAC = "adults+adolescents+children" vaccination strategy) and age group assuming 40 (baseline), 20 and 10 seeding imported infectors. **B** As **A**, but for the incidence of hospitalisations. **C** As **A**, but for the incidence of ICU admissions. **D** As **A**, but for the incidence of deaths. The horizontal dotted lines in **A**, **B**, and **D** respectively represent the rates of symptomatic cases, hospitalisations, and deaths of the first pandemic wave of COVID-19 in Wuhan, China^39^.

- 1. **Generation time**

To evaluate the robustness of our results to the assumed value of the generation time, we recalibrated the transmission rate ($\beta$) for the alternative value of generation time (7 days vs. 4.6 days assumed in the baseline analysis). We then applied all other conditions as in the baseline scenario. The resulting disease burden are insensitive to generation time across vaccination strategies and age groups (Figure S7).

**
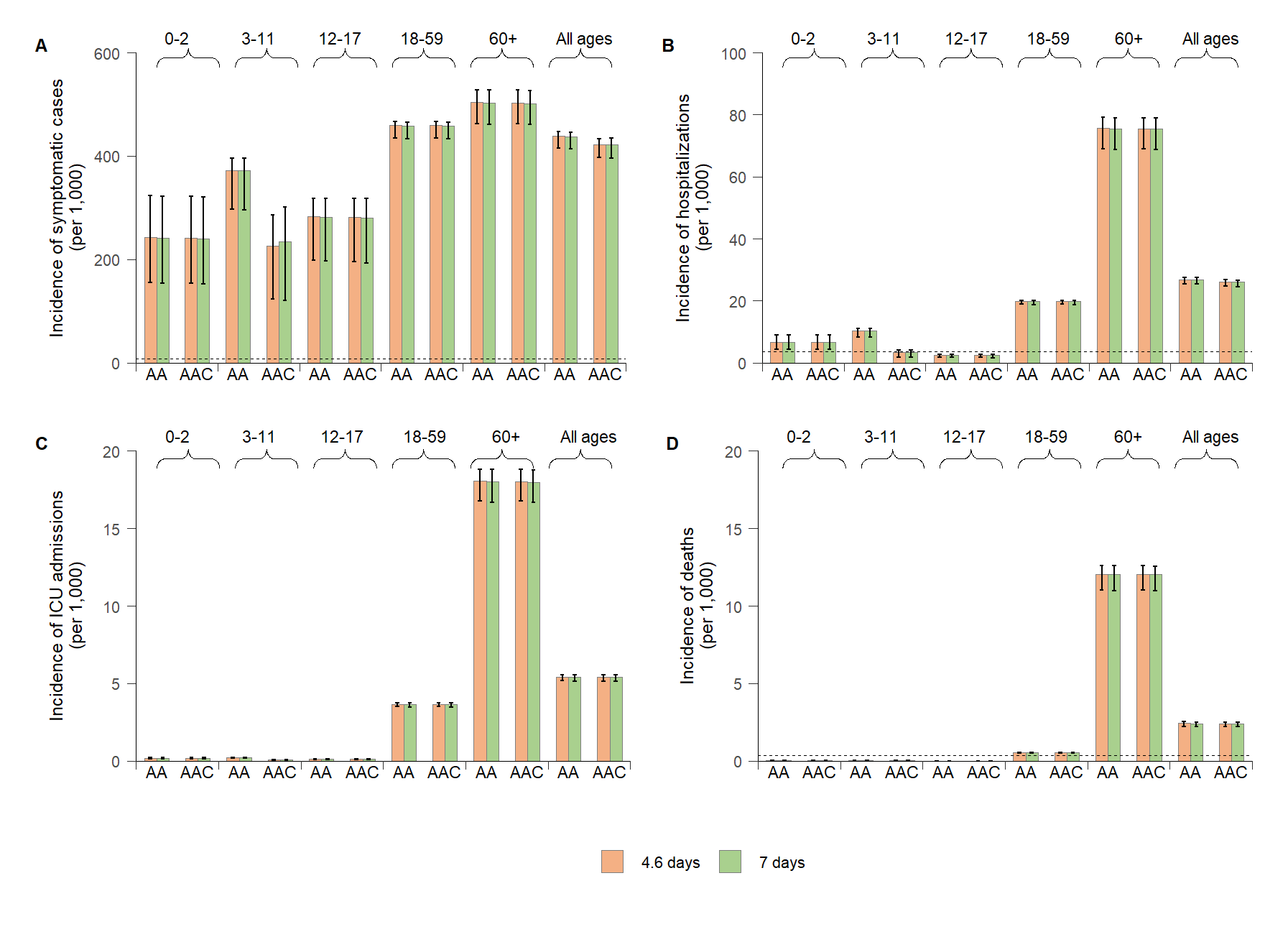
**

**Figure S7. Disease burden due to SARS-CoV-2 Delta variant infections in China assuming alternative generation time. A** Cumulative number of symptomatic cases per 1,000 individuals after 6 simulated months by vaccination strategy (AA = "adults+adolescents" vaccination strategy, AAC = "adults+adolescents+children" vaccination strategy) and age group assuming a generation time of 4.6 (baseline) or 7 days. **B** As **A**, but for the incidence of hospitalisations. **C** As **A**, but for the incidence of ICU admissions. **D** As **A**, but for the incidence of deaths. The horizontal dotted lines in **A**, **B**, and **D** respectively represent the rates of symptomatic cases, hospitalisations, and deaths of the first pandemic wave of COVID-19 in Wuhan, China^39^.

1. **Additional results**

**
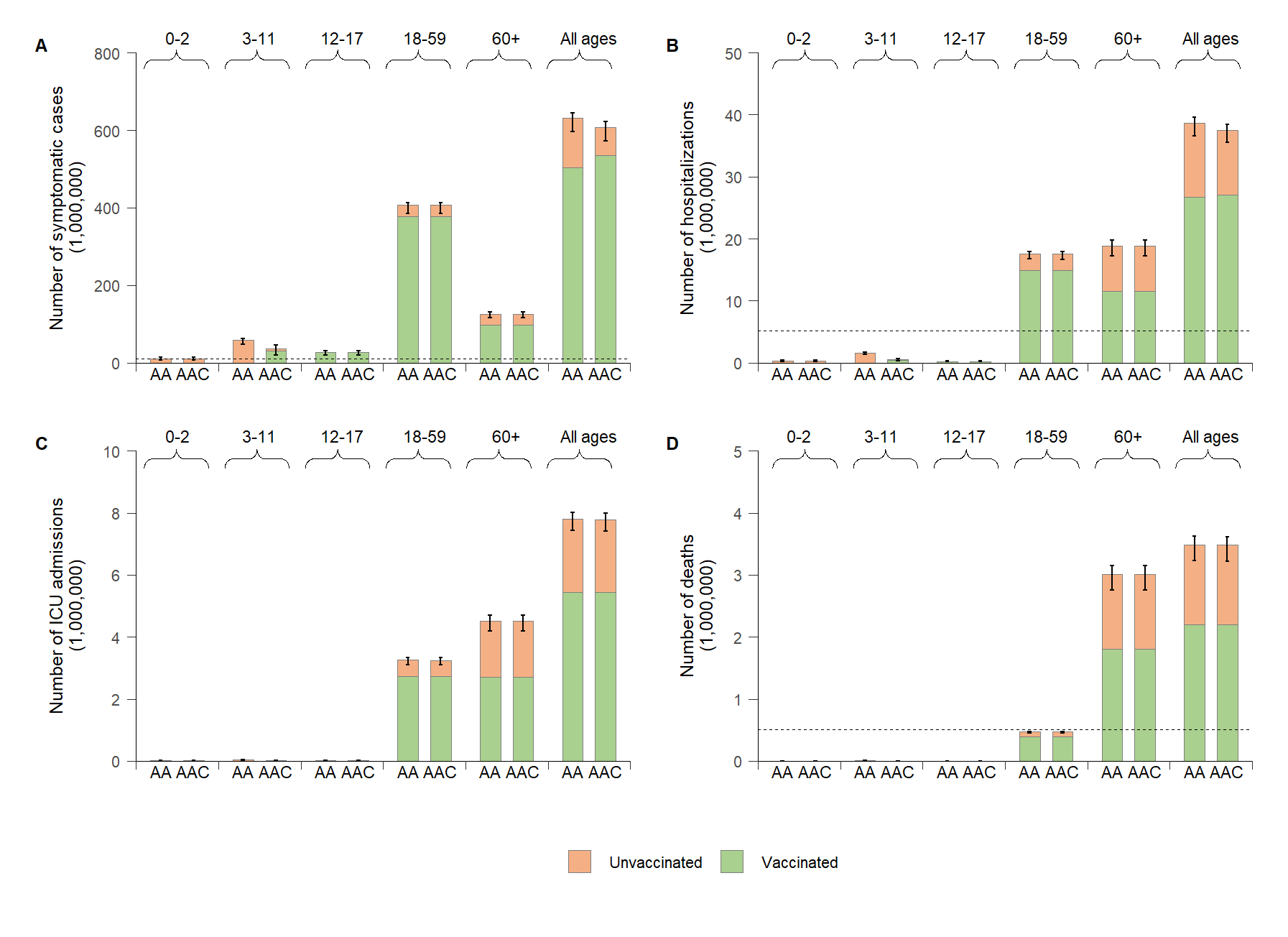
**

**Figure S8. Cumulative number of symptomatic cases, hospitalisations, ICU admissions, and deaths due to SARS-CoV-2 Delta variant infections in China under the baseline scenario. A** Cumulative number of symptomatic cases after 6 simulated months by vaccination strategy (AA = "adults+adolescents" vaccination strategy, AAC = "adults+adolescents+children" vaccination strategy), vaccination status, and age group. The vaccinated group are those individuals who have administered two doses. **B** As **A**, but for the number of hospitalisations. **C** As **A**, but for the number of ICU admissions. **D** As **A**, but for the number of deaths. The horizontal dotted lines in **A**, **B**, and **D** respectively represent the number of symptomatic cases, hospitalisations, and deaths that would occur in China based on the corresponding rates from the first pandemic wave of COVID-19 in Wuhan, China^39^.

**
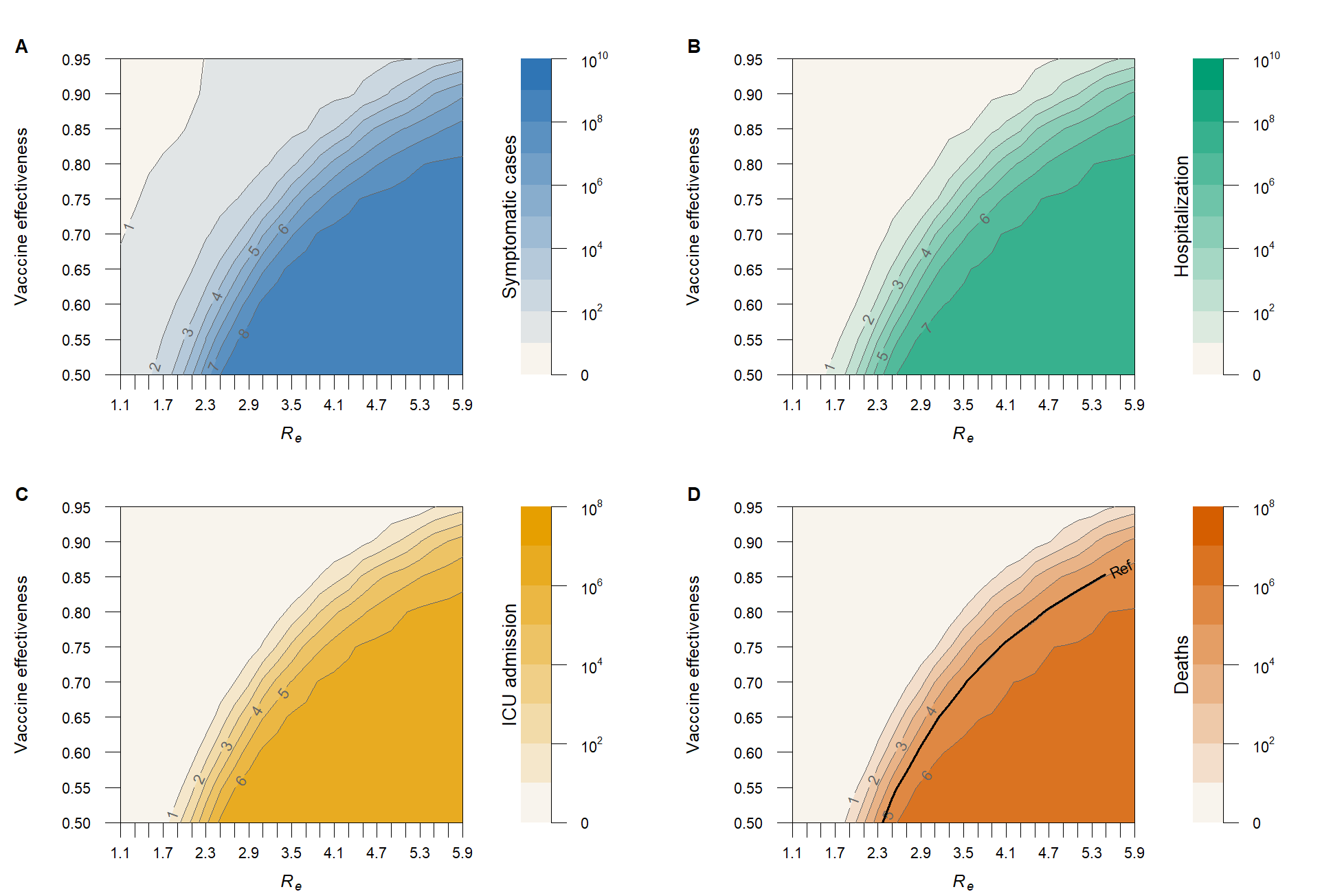
**

**Figure S9. Impact of adopting NPIs (*R_e_*) and increasing vaccine effectiveness (VE) against infections on disease burden due to SARS-CoV-2 Delta variant infections in China under the "adults+adolescents+children" vaccination strategy. A** Cumulative number of symptomatic cases (on a logarithmic scale) after 6 simulated months as a function of *R_e_* and VE. **B** As **A**, but for the cumulative number of hospitalisations. **C** As **A**, but for the cumulative number of ICU admissions. **D** As **A**, but for the cumulative number of deaths. The solid black reference line in **D** indicates the annual mean of 88,100 influenza-associated excess respiratory deaths in China^42^.

**References**

1. Zhang J, Klepac P, Read JM, et al. Patterns of human social contact and contact with animals in Shanghai, China. *Scientific Reports* 2019; **9**(1): 15141.

2. Hu S, Wang W, Wang Y, et al. Infectivity, susceptibility, and risk factors associated with SARS-CoV-2 transmission under intensive contact tracing in Hunan, China. *Nature Communications* 2021; **12**(1): 1533.

3. Al Kaabi N, Zhang Y, Xia S, et al. Effect of 2 Inactivated SARS-CoV-2 Vaccines on Symptomatic COVID-19 Infection in Adults: A Randomized Clinical Trial. *JAMA* 2021.

4. Palacios R, Batista AP, Albuquerque CSN, et al. Efficacy and safety of a COVID-19 inactivated vaccine in healthcare professionals in Brazil: the PROFISCOV study. *SSRN* 2021.

5. Han B, Song Y, Li C, et al. Safety, tolerability, and immunogenicity of an inactivated SARS-CoV-2 vaccine (CoronaVac) in healthy children and adolescents: a double-blind, randomised, controlled, phase 1/2 clinical trial. *The Lancet Infectious Diseases* 2021.

6. Kustin T, Harel N, Finkel U, et al. Evidence for increased breakthrough rates of SARS-CoV-2 variants of concern in BNT162b2-mRNA-vaccinated individuals. *Nature Medicine* 2021.

7. CDC. Improving communications around vaccine breakthrough and vaccine effectiveness 2021-07-29 2021. <https://context-cdn.washingtonpost.com/notes/prod/default/documents/8a726408-07bd-46bd-a945-3af0ae2f3c37/note/57c98604-3b54-44f0-8b44-b148d8f75165>. (accessed 2021-08-20 2021).

8. Halloran ME, Longini IM, Struchiner CJ, Longini IM. Design and analysis of vaccine studies: Springer; 2010.

9. Wang Z, Muecksch F, Schaefer-Babajew D, et al. Naturally enhanced neutralizing breadth against SARS-CoV-2 one year after infection. *Nature* 2021; **595**(7867): 426-31.

10. Zhang J, Litvinova M, Liang Y, et al. The impact of relaxing interventions on human contact patterns and SARS-CoV-2 transmission in China. *Science Advances* 2021; **7**(19): eabe2584.

11. Liu H, Zhang J, Cai J, et al. Investigating vaccine-induced immunity and its effect in mitigating SARS-CoV-2 epidemics in China. *BMC Medicine* 2022; **20**(1): 37.

12. Yang J, Marziano V, Deng X, et al. Despite vaccination, China needs non-pharmaceutical interventions to prevent widespread outbreaks of COVID-19 in 2021. *Nature Human Behaviour* 2021; **5**(8): 1009-20.

13. Kissler SM, Fauver JR, Mack C, et al. Viral Dynamics of SARS-CoV-2 Variants in Vaccinated and Unvaccinated Persons. *New England Journal of Medicine* 2021; **385**(26): 2489-91.

14. Riemersma KK, Grogan BE, Kita-Yarbro A, et al. Shedding of Infectious SARS-CoV-2 Despite Vaccination. *medRxiv* 2021: 2021.07.31.21261387.

15. Public Health England. SARS-CoV-2 variants of concern and variants under investigation in England - Technical briefing 20. 2021-08-06 2021. <https://assets.publishing.service.gov.uk/government/uploads/system/uploads/attachment_data/file/1009243/Technical_Briefing_20.pdf> (accessed 2021-08-28 2021).

16. Liu Q-H, Ajelli M, Aleta A, Merler S, Moreno Y, Vespignani A. Measurability of the epidemic reproduction number in data-driven contact networks. *Proceedings of the National Academy of Sciences* 2018; **115**(50): 12680.

17. Meng Z, Jianpeng X, Aiping D, et al. Transmission Dynamics of an Outbreak of the COVID-19 Delta Variant B.1.617.2 — Guangdong Province, China, May–June 2021. *China CDC Weekly* 2021; **3**(27): 584-6.

18. Hart WS, Miller E, Andrews NJ, et al. Generation time of the alpha and delta SARS-CoV-2 variants: an epidemiological analysis. *The Lancet Infectious Diseases* 2022.

19. Pung R, Mak TM, Kucharski AJ, Lee VJ. Serial intervals in SARS-CoV-2 B.1.617.2 variant cases. *The Lancet* 2021.

20. Sun K, Wang W, Gao L, et al. Transmission heterogeneities, kinetics, and controllability of SARS-CoV-2. *Science* 2020: eabe2424.

21. Li X-N, Huang Y, Wang W, et al. Effectiveness of inactivated SARS-CoV-2 vaccines against the Delta variant infection in Guangzhou: a test-negative case–control real-world study. *Emerging Microbes & Infections* 2021; **10**(1): 1751-9.

22. Kang M, Yi Y, Li Y, et al. Effectiveness of Inactivated COVID-19 Vaccines Against Illness Caused by the B.1.617.2 (Delta) Variant During an Outbreak in Guangdong, China. *Annals of Internal Medicine* 2022.

23. National Health Commission. COVID-19 vaccination status (as of February 1, 2022). 2022-02-01 2021. <http://www.nhc.gov.cn/xcs/yqjzqk/list_gzbd.shtml> (accessed 2022-03-18 2022).

24. The State Council Information Office PRC. Press conference held on situation regarding prevetion and control of the COVID-19 epidemic and vaccination. 2021-10-30 2021. <http://www.gov.cn/xinwen/gwylflkjz170/index.htm> (accessed 2021-03-19 2022).

25. National Health Commission. COVID-19 vaccination status. 2021-11-16 2021. <http://www.nhc.gov.cn/xcs/yqjzqk/list_gzbd.shtml> (accessed 2021-11-16 2021).

26. Wang Y, Chen R, Hu F, et al. Transmission, viral kinetics and clinical characteristics of the emergent SARS-CoV-2 Delta VOC in Guangzhou, China. *EClinicalMedicine* 2021; **40**.

27. Sheikh A, McMenamin J, Taylor B, Robertson C. SARS-CoV-2 Delta VOC in Scotland: demographics, risk of hospital admission, and vaccine effectiveness. *The Lancet* 2021.

28. Twohig KA, Nyberg T, Zaidi A, et al. Hospital admission and emergency care attendance risk for SARS-CoV-2 delta (B.1.617.2) compared with alpha (B.1.1.7) variants of concern: a cohort study. *The Lancet Infectious Diseases* 2021.

29. Fisman DN, Tuite AR. Evaluation of the relative virulence of novel SARS-CoV-2 variants: a retrospective cohort study in Ontario, Canada. *Canadian Medical Association Journal* 2021; **193**(42): E1619.

30. Nyberg T, Twohig KA, Harris RJ, et al. Risk of hospital admission for patients with SARS-CoV-2 variant B.1.1.7: cohort analysis. *BMJ* 2021; **373**: n1412.

31. Graham MS, Sudre CH, May A, et al. Changes in symptomatology, reinfection, and transmissibility associated with the SARS-CoV-2 variant B.1.1.7: an ecological study. *The Lancet Public Health* 2021; **6**(5): e335-e45.

32. Antonelli M, Penfold RS, Merino J, et al. Risk factors and disease profile of post-vaccination SARS-CoV-2 infection in UK users of the COVID Symptom Study app: a prospective, community-based, nested, case-control study. *The Lancet Infectious Diseases* 2021.

33. Han S, Cai J, Yang J, et al. Time-varying optimization of COVID-19 vaccine prioritization in the context of limited vaccination capacity. *Nature Communications* 2021; **12**(1): 4673.

34. Dan W, Yanyang Z, Lin T, et al. Effectiveness of Inactivated COVID-19 Vaccines Against Symptomatic, Pneumonia, and Severe Disease Caused by the Delta Variant: Real World Study and Evidence — China, 2021. *China CDC Weekly* 2021; **4**(4): 57-65.

35. Cerqueira-Silva T, Katikireddi SV, de Araujo Oliveira V, et al. Vaccine effectiveness of heterologous CoronaVac plus BNT162b2 in Brazil. *Nature Medicine* 2022.

36. Heath PT, Galiza EP, Baxter DN, et al. Safety and Efficacy of NVX-CoV2373 Covid-19 Vaccine. *New England Journal of Medicine* 2021.

37. United Nations. World Population Prospects 2019. 2019-08-28 2021. <http://www.cansinotech.com.cn/html/1///179/180/806.html> (accessed 2021-04-19 2021).

38. Viner RM, Mytton OT, Bonell C, et al. Susceptibility to SARS-CoV-2 Infection Among Children and Adolescents Compared With Adults: A Systematic Review and Meta-analysis. *JAMA Pediatrics* 2021; **175**(2): 143-56.

39. Yang J, Chen X, Deng X, et al. Disease burden and clinical severity of the first pandemic wave of COVID-19 in Wuhan, China. *Nature Communications* 2020; **11**(1): 5411.

40. Zhang J, Litvinova M, Liang Y, et al. Changes in contact patterns shape the dynamics of the COVID-19 outbreak in China. *Science* 2020: eabb8001.
